# Dysregulation in level of goal and action identification across psychological disorders

**DOI:** 10.1016/j.cpr.2010.05.004

**Published:** 2011-03

**Authors:** Edward Watkins

**Affiliations:** University of Exeter, United Kingdom

**Keywords:** Control theory, Action identification, Rumination, Transdiagnostic, Impulsive, Problem solving, Abstract, Concrete

## Abstract

Goals, events, and actions can be mentally represented within a hierarchical framework that ranges from more abstract to more concrete levels of identification. A more abstract level of identification involves general, superordinate, and decontextualized mental representations that convey the meaning of goals, events, and actions, “why” an action is performed, and its purpose, ends, and consequences. A more concrete level of identification involves specific and subordinate mental representations that include contextual details of goals, events, and actions, and the specific “how” details of an action. This review considers three lines of evidence for considering that dysregulation of level of goal/action identification may be a transdiagnostic process. First, there is evidence that different levels of identification have distinct functional consequences and that in non-clinical samples level of goal/action identification appears to be regulated in a flexible and adaptive way to match the level of goal/action identification to circumstances. Second, there is evidence that level of goal/action identification causally influences symptoms and processes involved in psychological disorders, including emotional response, repetitive thought, impulsivity, problem solving and procrastination. Third, there is evidence that the level of goal/action identification is biased and/or dysregulated in certain psychological disorders, with a bias towards more abstract identification for negative events in depression, GAD, PTSD, and social anxiety.

## 1 Introduction

In an early review of the utility of a transdiagnostic or “across disorder” perspective, Harvey, Watkins, Mansell and Shafran (2004) concluded that there was evidence consistent with a number of definite transdiagnostic cognitive and behavioral processes (e.g., selective attention, recurrent thinking, and avoidance behaviour), which can be found across different disorders. One reason to expect such processes to be common across disorders is that these processes (e.g., selective attention) may reflect normal psychological operations that are present in non-pathological individuals but which become suborned to dysfunctional uses or exaggerated as pathology develops. Building on this rationale, the current review proposes that dysregulation in level of identification for goals, actions, events, and outcomes is a possible transdiagnostic process that may be important across a range of disorders.

The hypothesis that goals, events, and actions can be mentally represented within a hierarchical framework that ranges from more abstract to more concrete levels of identification is an important concept within a number of social–cognitive theories (e.g., control theory, Carver & Scheier, 1982, 1990; Powers, 1973a; construal level theory, Trope & Liberman, 2003; action identification theory, Vallacher & Wegner, 1987). The conceptualization of abstract versus concrete that is common to all of these theories is that a more abstract level of identification involves general, superordinate, and decontextualized mental representations that convey the essential meaning of goals, events, and actions that denote the “why” aspects of an action including the ends that are consequential to it. In contrast, a more concrete level of identification involves subordinate, contextual, and specific details of goal, events, and actions that denote the feasibility, mechanics, and means of “how” to do the action. Control theory approaches hypothesize that goals, events, and behaviors are hierarchically organized and can be processed at different levels of abstraction, with more abstract, superordinate goals and standards guiding and informing more concrete, subordinate goals and standards (Broadbent, 1977; Carver & Scheier, 1982, 1990, 1998; Emmons, 1992; Powers, 1973a, b). Similarly, within Action Identification Theory (AIT; Vallacher & Wegner, 1987), an abstract identification represents the purpose and ends of an action (“why it is carried out”), whereas a concrete identification represents the action's process and means (“how it is carried out”). Thus, within AIT, the same action (e.g., “locking my door”) can be identified in abstract terms (e.g., “maintaining security”, the purpose why the door is locked, i.e., a higher level in the goal hierarchy) or in more concrete terms (e.g., “turning a key”, the means how the action is implemented, i.e., a lower level in the goal hierarchy). Abstract identifications of goals, events, and behaviors are focused on the desirability and importance of outcomes, whereas concrete identifications are focused on the feasibility, mechanics, and planning of outcomes. Importantly, clinically-relevant theories have proposed that the level of abstraction used to represent goals, behaviors, and events may be an important dimension that influences psychopathology (e.g., the reduced concreteness theory of worry, Borkovec, Ray, & Stöber, 1998; the processing mode theory, Watkins, 2008; theories of escape from self, Baumeister, 1990; Heatherton & Baumeister, 1991).

This paper develops these ideas concerning the role of the level of abstraction used to represent goals, behaviors, and events (henceforward referred to as *level of goal/action identification*^1^) in the onset and maintenance of psychopathology by developing a theoretical analysis grounded within a control theory approach (Carver & Scheier, 1982; Powers, 1973a; Vallacher & Wegner, 1987; Watkins, 2008), and then examining the evidence relevant to this analysis. To anticipate the main hypothesis, the current analysis proposes that dysregulation of goal/action identification, that is, an inability to flexibly shift between an abstract level of goal/action identification, focused on higher-level ends, and a concrete level of goal/action identification, focused on lower-level means, is a transdiagnostic process underpinning psychopathology. This hypothesis is based on three lines of evidence. First, different levels-of-goal/action identification have distinct functional effects on a range of cognitive, emotional, and behavioral outcomes that are symptoms of psychological disorders and/or processes implicated in psychopathology (e.g., emotional reactivity, Watkins, Moberly & Moulds, 2008; self-control, Fujita, Trope, Liberman & Levin-Sagi, 2006; repetitive thought, Watkins, 2008). Second, the optimal level of goal/action identification depends upon specific circumstances, and level of goal/action identification shifts adaptively in response to context, including difficulty (Vallacher & Wegner, 1987) and mood state (Forgas, 2008), suggesting that level of goal/action identification is functionally regulated in non-disordered individuals. Third, the predominant level of goal/action identification adopted in certain psychological disorders appears to be abnormal relative to controls. Thus, there is evidence suggesting that level of goal/action identification can influence outcomes relevant to psychological disorders and that regulation of level of goal/action identification is a normal and adaptive process found in healthy individuals. As such, it is a plausible hypothesis that disturbances in this process might be found across a range of psychological disorders and contribute to the onset and maintenance of those disorders. This review will examine the theories and evidence relevant to the regulation of level of goal/action identification, including evidence that it is regulated in response to change in circumstances, and review its functional effects, before examining evidence that such regulation of level of goal/action identification may be impaired in different psychological disorders, including evidence of asymmetric bias towards abstract or concrete identification within particular disorders.

## 2 The underpinning theories: control theory and action identification theory

The current analysis is theoretically based within control theory and action identification accounts, using the principles of these theories to generate the hypotheses examined in the review. A brief review of these theories, their key principles and predictions is therefore necessary.

Control theory provides an integrative theoretical account of human behavior, with particular reference to self-regulation, motivation, and emotional experience (see Carver & Scheier, 1982; Higginson, Mansell, & Wood, in press; Powers, 1973a). A key element within control theory accounts is the negative feedback loop, which functions to reduce perceived deviations from a reference value, which corresponds to an individual's goal or purpose. The reference value is compared against perceptual input, and any discrepancy (or error) between the present state perceived and the reference value generates a behavior directed towards the goal specified in the reference value, with the function of reducing the discrepancy. The behavior does not act to reduce the discrepancy directly but by having an impact on the environment, which in turn changes the perceptual input into the feedback loop. Thus, critically the function of the negative feedback loop is not to generate behavior but rather to set up and maintain conditions in which the perceptual input matches the reference value, that is, to control its perception of the environment, and, thereby minimise discrepancy.

A second key element within control theory accounts is that these reference values or goals can be arranged in a hierarchy of means and ends in which subordinate, concrete goals, or means, serve to achieve the more abstract, superordinate goals, or ends. In control theory accounts (Carver & Scheier, 1982, 1990, 1998; Emmons, 1992; Powers, 1973a,b), this hierarchical organization of goals is represented by a hierarchical organization of negative feedback loops. Within this hierarchical organization, pursuit towards abstract goals occurs by specifying reference values at the next lower level of abstraction, all the way down to the concrete representations required to specify the actual behaviors needed to progress towards the goal. For example, one classification of levels within control theory approaches (Carver & Scheier, 1990; Powers, 1973a) proposes that the reference values at the most abstract levels may represent a global sense of idealized self (i.e., a decontextualized, superordinate meaning capturing the essence of the self), which in turn sets the broad principles that organize goals and behavioral standards across multiple situations (e.g., to be an honest person), whereas the reference values at the more concrete levels represent the specific actions and behavioral programs and sequences necessary to implement the principles in a particular situation (e.g., telling the truth to a friend, i.e., more contextualized, specific details of how to do the action). From this conceptualization, it can be seen that the reference values and associated perceptual input within control theory can include representations of both implications and meanings for the self, as well as specific behaviors, events, and outcomes.

Third, control theory accounts propose that a particular level in the hierarchy may be functionally and operationally prepotent and superordinate at any moment in time reflecting whether the individual is focusing attention and awareness on a more abstract or concrete level, and thereby representing reference values (goals) and perceptual signals (from the environment) in a more abstract or concrete manner (i.e., this corresponds to the current predominant level of goal/action identification; Carver & Scheier, 1982; Vallacher & Wegner, 1987). It has been hypothesized that the “highest level of control operating at any given moment corresponds to the level at which the person is focally attentive at that moment” (Carver & Scheier, 1982, p. 117), that is, whether the individual is focusing on a more abstract or more concrete level of processing. Not every level of control needs to be involved in all acts of self-regulation: People may often function at the level of program control with little or no reference to higher-order goals. Alternatively, self-regulation can occur at a highly abstract level if the behavior is relatively familiar and well-practised, because all subordinate levels would continue to operate because their operation occurs in service of the functionally superordinate level.

Fourth, control theory accounts hypothesize that effective self-regulation (i.e., effective reduction of discrepancy) requires flexible and balanced coordination between the different levels within the goal hierarchy, such that the superordinate level of control adaptively varies in response to situational and task demands. There are two elements to this effective coordination. Firstly, because pursuit towards abstract goals occurs by specifying reference values at the next lower level of abstraction, effective self-regulation requires that abstract levels in the hierarchy are functionally connected and engaged with concrete levels, with all interfacing with the present environment. Breakdown of specification from higher levels to lower levels in the hierarchy, whether through mismatch of reference values or a lack of procedural detail with respect to behavioral programs and sequences, will impair effective self-regulation (see Section 3.1 for further detail). Secondly, the prepotent level of goal/action identification (i.e., where awareness is currently located in the hierarchy) needs to be shifted to match the circumstances. Critically, depending on context, a level of control that is too abstract, too concrete or that fails to link abstract levels to concrete levels is hypothesized to be detrimental (Carver & Scheier, 1998, chapter 13).

Thus, Action Identification Theory (AIT: Vallacher & Wegner, 1987; Vallacher, Wegner & Somoza, 1989) hypothesizes that there is an optimal level of action identification that varies with circumstances such that performance is improved and negative emotional response lessened when a personally easy action is identified in relatively abstract terms (the action's purpose and meaning) or when a personally difficult, complex, or unfamiliar action is identified in relatively concrete terms (the action's process and mechanical details). AIT proposes that “when both a lower and a higher level of identity are available, there is a tendency for the higher-level identity to become prepotent” (Vallacher & Wegner, 1987, p.5). In other words, by default, individuals will tend to adopt a more abstract level of identification and be sensitive to the broader meanings and implications of what they are doing. Consistent with this, there is evidence that when a person has identified his or her current behavior at a concrete level, there is greater readiness to accept a more abstract identity made available by the context surrounding the action than if the behavior was already identified at an abstract level, with the emergence of this new identity promoting new courses of action (Wegner, Vallacher, Kiersted, & Dizadji, 1986; Wegner, Vallacher, Macomber, Wood & Arps, 1984). However, AIT also hypothesizes that “when an action cannot be maintained in terms of its prepotent identity, there is a tendency for a lower level identity to become prepotent” (Vallacher & Wegner, 1987, p.5). Thus, AIT proposes that when faced with difficulties in performing an action, individuals shift to more concrete levels of identification in order to focus on *how* to perform the action, thereby grounding them back in the realities of the current environment. AIT thus proposes that the level of goal/action identification adopted is dynamically adjusted, oscillating from more abstract to more concrete levels within the hierarchy in response to circumstances, until eventually over repeated actions, the individual converges on the level of identification that enables him or her to perform that particular behavior up to his or her capacity. AIT predicts that an action will be identified at a more abstract level to the extent that it is seen as easy to do, familiar, can only be enacted in a limited number of ways, is short in duration, and requires little time to learn well, such that well-practised actions will tend to be represented more abstractly, consistent with the circumstances when there is good integration down from abstract levels to concrete levels.

Because the terms abstract and concrete are used multifariously (and often vaguely) in psychology (and in lay speech), it is useful to define exactly what is meant by abstract versus concrete identifications in the current analysis. As the earlier paragraphs make clear, the current analysis is grounded within control theory, and as such, the current analysis operationalizes more abstract identifications as those representing superordinate goals including the purpose, meanings, and ends of a goal or action (“why it is carried out”), and more concrete identifications as those representing subordinate goals including the process and means of a goal or action (“how it is carried out”). Level of goal/action identification was chosen as the operationalization of abstraction in the current theoretical framework because: (a) the current analysis is grounded within control theory accounts and level of goal/action identification is the primary feature of abstract versus concrete processing within this theoretical approach; (b) this is an element of abstraction for which there are clear theoretical accounts of how it may influence psychopathology and be responsive to context (Carver & Scheier, 1990; Vallacher & Wegner, 1987; Watkins, 2008); c) means versus ends is the operationalization of abstractness that is common to all the theoretical perspectives considered; and d) there are advantages in clarity, parsimony, and theoretical rigor in adopting a narrow and specific operationalization of abstraction.

Moreover, it is important to be clear that the current analysis concentrates on the level of identification that is the functionally superordinate and prepotent operational level at any moment, and how this level of prepotent identification can shift in response to circumstances, with distinct consequences (with the phrase “level of goal/action identification” used as shorthand to refer to the level of identification of goal, actions, and events that is currently functionally prepotent in the hierarchy). Thus, the current analysis focuses on one implication derived from control theory approaches concerning the effect of shifting the current prepotent level of goal/action identification on psychopathology. Indeed, a full consideration of all implications emerging from control theory for psychopathology is beyond the scope of the current article (see Higginson et al., in press for a discussion of some other implications). Nonetheless, it is important to distinguish the currently prepotent level of goal/action identification and its functions from the ongoing representations within each level of the hierarchy and their dynamic interactions. The current analysis is focused on the ability to make momentary shifts in the prepotent level of identification in response to circumstances, and the implications of such (in)flexibility for psychopathology. This is distinct from changes within the actual content of the representations at each level of identification, which is, of course, a natural consequence of the operation of the feedback loops, both within and across levels — as behavior causes changes in the environment that signals movement towards or away from a reference value, the reference values fed down to subordinate levels may change. Moreover, as Carver and Scheier (1982, p.117) note “reference values in the hierarchy are being matched (and new values being substituted) more quickly at lower levels that at higher levels. That is, many changes in muscle tensions are involved in making a single turn; it takes many turns to get to the friend's house; and it takes more than one act of responsibility to sustain one's self-esteem.” Thus, changes *within* the content of concrete levels of identification occur on short-term scales and are more visible to observers, whereas changes *within* the content of abstract levels of identification take longer, are less frequent and are less visible but have more enduring impact for the individual because they influence the setting of all the subordinate levels of the hierarchy. Moreover, studies examining AIT have demonstrated that the content of both concrete and abstract identifications can be responsive to environmental context resulting in the integration of existing elements into new structures *within* that level of identification (Wegner et al., 1986, 1984). Such ongoing changes *within* the representations of reference values at different levels within the hierarchy in response to feedback are clearly an important part of the process of self-regulation. For example, Perceptual Control Theory (Powers, 1973a) proposes that psychopathology is a consequence of conflicting goals, and that reorganization of goal structure (resetting of sub-goals) through a trial-and-error learning process is how this conflict and associated difficulties are resolved. However, this is distinct from shifts *between* a prepotent concrete level of identification to a prepotent abstract level of identification (or vice versa), which is the focus of the current analysis (although as discussed in Section 3.1, shifts in the prepotent level of identification may influence the process of changes within reference values).

## 3 Flexible regulation of level of goal/action identification

### 3.1 Functional effects of different levels of goal/action identification

The control theory accounts hypothesize that level of goal/action identification shifts in response to circumstances in a functional way, and, thus, as a corollary, dysregulation of level of goal/action identification may be problematic. Both AIT and control theory propose that the level of goal/action identification adopted is adaptive when it matches the function required of the task in hand. Within these theories, the potential advantages and disadvantages of an abstract versus concrete level of identification centre around their relative sensitivity to contextual and situational detail. Relative to a concrete level of identification, an abstract identification insulates an individual from the specific context, making the individual less distractible, less impulsive, and enabling more consistency and stability of goal pursuit across time, but also making the individual less responsive to the environment and to any situational change, and providing fewer specific guides to action and problem solving because of their distance from the mechanics of action (see Table 1 for summary of conditions under which different levels of goal/action identification have distinct consequences).

Thus, adopting an abstract level of identification is hypothesized to have the following advantages: (a) it increases consistency and stability of behavior towards long-term goals across time and across different situational demands, because it ensures that subordinate goals and actions remain directed towards personally important goals and minimizes interference from incidental influences (Carver & Scheier, 1998; Fujita et al., 2006; Vallacher & Wegner, 1987, 1989); (b) it provides more flexibility in responding to relatively concrete goals that are unattained, because an abstract level of identification affords more alternative sub-goals and behaviors to resolve the goal discrepancy (Brunstein & Gollwitzer, 1996, e.g., for poor progress on the goal of writing a poem, identifying the goal abstractly as “to be creative” provides alternative routes to the goal such as playing music); (c) it facilitates the resolution of chronic goal conflict, in which there are two incompatible sub-goals (e.g., “always tell the truth”, “don't upset other people”), generated from the same higher-order goal (e.g., be a considerate person), which is hypothesized to be a cause of psychological distress (Powers, 1973a). Processing at a more abstract level of identification can resolve such goal conflict either by affording alternative subordinate sub-goals to serve the abstract goal that are not in conflict (i.e., flexibility as above) or by adjusting the importance of the sub-goals (i.e., ends and consequences) so as to prioritize one over the other. Thus, in the main, adopting a more abstract level of identification (i.e., having a higher level in the goal hierarchy as the prepotent operational level) will be advantageous.

However, there are circumstances when a concrete level of identification is hypothesized to be adaptive and an abstract level of identification maladaptive. First, because pursuit towards abstract goals occurs by specifying reference values at the next lower level, down to the actual concrete behaviors required, an abstract level of identification will only be advantageous when there is extensive and relevant procedural knowledge specifying the links between the abstract purpose and the concrete means necessary to achieve it, such as when an activity is well-practised or straightforward. Under circumstances of complexity, unfamiliarity, difficulty, or stress, this specification of reference values down through the control hierarchy breaks down, and the advantages of abstract construal are lost. Thus, as proposed by Carver and Scheier (1982, p.125) “it is likely that a good deal of the behavioral disruption that is viewed as neurotic or maladaptive stems from an inability to specify reference values from the level of system concepts (or principles) down to and through the level of program control.” For example, adopting an abstract level of identification as prepotent would not be useful for a learner driver still getting used to handling a car or for an experienced driver in hazardous, unfamiliar driving conditions such as a snowstorm, since in both cases there is not well-established specification of how abstract reference values translate into sub-goals and concrete behavior. Instead, in these circumstances, self-regulation needs to be guided by a more concrete level of goal/action identification that serve the function of determining the specific means and actions (*how*) by which to best proceed and focusing close attention on the immediate environment. A similar point is made by Powers (1973a) when he notes that higher-level control systems can be functionally dissociated from lower level system, to operate within the ‘imagination mode’, in which mode the higher-level control systems can become disengaged from the present moment, with the potential for maladaptive consequences.

Within control theory terms, the circumstances considered difficult can be operationalized as when progress towards a goal occurs at a rate lower than the standard (or is expected to occur at a rate lower than desired), as detected by a “meta-monitoring system”, which is a further negative feedback loop checking on how well a control feedback loop is reducing the discrepancy between its particular reference value and perceptual input (Carver & Scheier, 1990). Importantly, when the rate of progress at reducing the discrepancy in the action loop is slower than the meta-monitoring system's reference value, doubt and negative affect result.^2^ Thus, a concrete level of goal/action identification may be expected to be more adaptive when there is a slower rate of progress than expected on personally relevant goals, which may be signalled by negative affect and/or negative cognition. Within this analysis, insufficient rate of goal progress (difficulty) can be further operationalized as resulting from: a) a lack of specification from abstract levels to concrete levels (i.e., an unfamiliar situation or a complex task that is not yet well-practised); b) incorrect specification of reference values down the hierarchy (i.e., a mismatch where the reference value at a concrete level is irrelevant or unhelpful for the higher-level goal for the higher-level goal; c) disturbances and obstacles that impede or disrupt progress even when the procedural knowledge between abstract and concrete levels is well established, resulting from the environment (e.g., unusual conditions, sudden changes in situation, an unexpected response) or internal state (e.g., tiredness, mood, anxiety); and d) setting too high a reference standard for progress.

Second, when the superordinate abstract goal is ill-defined and it is difficult to specify how it might actually be achieved, adopting an abstract level of goal/action identification as prepotent is going to be problematic, since the subordinate levels will continue to operate in the service of whatever level is functionally superordinate but in the absence of a useful reference value (e.g., a goal like “be happy” is too vague to provide clear guidance as to how an individual might specify sub-goals towards attaining it). In contrast, adopting a concrete level of identification may help with goal progress by breaking the task down into more specific, proximal sub-goals and associated behavioral steps, and thereby, provide immediate direction to one's behavior and more discrete and salient markers of progress (Emmons, 1992), leading to improved self-efficacy, improved motivation, and improved task performance (Bandura & Schunk, 1981; Stock & Cervone, 1990). It is easier to determine if one is being successful at pursuing a lower-level goal like “keeping your desk clean” than the associated higher-level goal of “being more organized”.

Third, processing at a more abstract level may interfere with goal disengagement: the more abstract the level at which a goal is represented in the hierarchy, the more important the goal becomes to the general sense of self, and the harder it becomes to disengage from the goal (Martin & Tesser, 1989, 1996). When a goal is difficult or impossible to attain, processing at too abstract a level will make it harder to relinquish the goal, trapping the individual in the invidious state where he or she can neither make progress toward the goal nor abandon it, leading to ongoing negative affect and depression (Hamilton, Greenberg, Pyszczynski & Cather, 1993; Pyszczynski & Greenberg, 1987) and persistent unconstructive repetitive thought (Watkins, 2008).

Fourth, the level of goal/action identification may influence the emotional response to a rate of progress on a goal lower than the standard. Carver and Scheier (1990, p. 23) noted that “it seems that discrepancies noted by the meta system have greater emotional impact when they concern a central element of the self than when they bear only on a more peripheral goal (a program or a sequence of action). Sometimes a task failure has a big impact on one's feelings, sometimes not. The difference between these cases would seem to be the level of abstraction at which the person is focusing.” Thus, processing a negative outcome (poor goal progress) at a more abstract level of goal/action identification may intensify its negative emotional impact.

Carver and Scheier (1998) further proposed that the effects of adopting a more concrete level of identification will depend on whether the concrete level of identification is linked to making progress on an abstract goal higher in the hierarchy or is instead a means of disengaging attention from an abstract reference value. If a concrete identification focuses on the concrete means necessary to deliver a higher-level abstract goal, it will be adaptive in achieving the goal. However, if the concrete identification serves to protect the person from an awareness of more abstract consequences and implications, particularly concerning the self, it is less likely to facilitate goal progress.

This argument is functionally equivalent to the theory of “escape from self” (Baumeister, 1990; Heatherton & Baumeister, 1991), used to explain impulsive responses in eating disorders, alcohol use, and suicide, in which exclusive focus on concrete details and sensory experience, such as in a binge, can be a means to avoid any higher-level thought, such as abstract evaluations of the self. The theory of “escape from self” is explicit that dysregulation in level of goal/action identification may be a mechanism in the development of a suicide attempt or a binge. The escape theory argues that an aversive state of high self-awareness occurs from making internal attributions and unfavourable comparisons concerning discrepancies between desired state and current state, leading to negative evaluations and implications about the self (i.e., abstract level of goal identification involving abstract reference values and abstract perceptual signals), which leads to increased negative affect. In response to this negative affect and awareness of negative self-related implications, the individual attempts to escape from these negative evaluations by adopting concrete processing focused on immediate sensory experience. This concrete level of identification reduces inhibitions and impulsiveness, leading to an increased likelihood of attempting suicide, binging, drinking, drug use, or other impulsive behavior.

### 3.2 Adaptive regulation of level of goal and action identification

Thus, control theory and AIT generate the hypothesis that a concrete level of goal/action identification is typically adaptive under circumstances of complexity, unfamiliarity, difficulty, or stress (i.e., when there is not a well-developed connection from higher-level goals to lower-level goals or when prior specifications of reference values down the goal hierarchy is no longer appropriate because of changing circumstances), because it facilitates detailed guidance as to what to do next, whereas under circumstances that are safe, familiar, and straightforward (i.e., where there is a well-developed specification of reference values including a range of alternative sub-goals readily available) or require focus on long-term goals, abstract construal is hypothesized to be adaptive, as it enables stability and flexibility in goal pursuit (see Table 1). Moreover, since control theory accounts hypothesize that mood is a function of relative goal progress, with negative versus positive affect resulting from less versus greater progress towards a goal than desired and expected (Carver & Scheier, 1990), it is hypothesized that negative affect would be associated with a shift to a more concrete level of goal/action identification. Similar predictions have been made by theories that propose that affect influences the process of cognition (Bless & Fiedler, 2006; Fiedler, 2001; Forgas, 2008). These theories propose that positive affect promotes an abstract, global processing style, whereas negative affect promotes a concrete, local, processing style that focuses on the demands of the external world, paying careful attention to external stimulus information. Like AIT and control theory, this effect of mood on level of goal/action identification is hypothesized to be adaptive in that it matches the level of cognitive processing to situational need (Bless et al., 1996; Beukeboom & Semin, 2005; Forgas, 2008). If a negative mood signals a problematic situation, then a shift to a concrete level of goal/action identification would be adaptive because it provides more contextual detail about the specific means and alternatives by which to proceed, whereas abstract processing is more functional in safe, familiar situations, characterized by positive affect.

There is considerable evidence consistent with the hypotheses that: (a) different levels of goal/action identifications have distinct functional effects, with abstract identifications aiding long-term goal pursuit, and concrete identifications more adaptive in difficult circumstances; (b) level of goal/action identification shifts adaptively to circumstances. First, individuals tend by default to use a more abstract level of identification (Wegner & Vallacher, 1987; Wegner et al., 1986, 1984), yet when faced with difficult, novel or complex situations, people often move towards more concrete identifications (Vallacher, Wegner & Frederick, 1987; Wegner et al., 1984). For example, when given an unwieldy cup that made drinking more difficult, participants tended to provide more concrete descriptions of the act of drinking than individuals given normal cups (Wegner et al., 1984). Second, when giving a speech, focusing on the concrete process of giving the speech resulted in less anxiety and less performance dissatisfaction when the task was perceived to be difficult, whereas focusing on the abstract purpose of the speech resulted in less anxiety and less performance dissatisfaction when the task was perceived to be easy (Vallacher et al., 1989; Vallacher, Wegner, McMahan, Cotter & Larsen, 1992). Third, a concrete level of identification can facilitate effective behavioral self-regulation by improving problem solving (Watkins & Baracaia, 2002; Watkins & Moulds, 2005a), and by focusing attention on the immediate demands of the present situation, and freeing up cognitive resources (e.g., Gollwitzer, 1999; Webb & Sheeran, 2003). Fourth, a habitual tendency towards more abstract level of identification is associated with more persistent and stable behavior, greater self-motivation, less impulsiveness, and fewer action errors (Vallacher & Wegner, 1989). Fifth, inducing an abstract level of identification produces greater self-control on experimental tasks than inducing a concrete level of identification (Fujita et al., 2006).

Moreover, there is extensive evidence supporting the hypothesis that level of goal/action identification shifts with mood. Happy mood is associated with more global and abstract level of goal/action identification, whereas sad mood is associated with a more local, concrete level of goal/action identification that is more attentive to detailed, external information (e.g., Beukeboom & Semin, 2005, 2006; Bless et al., 1996; Forgas, 2007; Forgas & East, 2008; Gasper & Clore, 2002; Storbeck & Clore, 2005). For example, Beukeboom and Semin (2005) used the Behavioral Identification Form (BIF), which presents participants with a list of behaviors (e.g., “eating”), and asks them to endorse one of two subsequent descriptions differing in their level of action identification (e.g., “getting nutrition” [abstract], versus “chewing and swallowing” [concrete]). Participants in a happy mood endorsed descriptions of actions that were more abstract than participant in a sad mood.

Thus, there is evidence in non-clinical research samples that level of goal/action identification is flexibly regulated in response to circumstances, and, in particular, in response to mood state. Consistent with control theory and AIT, non-clinical participants adopt the level of goal/action identification hypothesized to be most adaptive to the current circumstances. Furthermore, there is evidence consistent with the hypotheses that different levels of goal/action identification have distinct functional effects. Taken together, the extant evidence suggests that level of goal/action identification is adaptively regulated to circumstances in healthy controls. Given the adaptive regulation of level of goal/action identification typically seen in healthy controls, it is a plausible hypothesis that psychological disorders may involve impaired regulation of level of goal/action identification.

## 4 Level of goal/action identification is causally implicated in symptoms and psychopathology

Moreover, any evidence that manipulating level of goal/action identification influences clinically-relevant outcomes, such as symptoms and processes involved in psychological disorders, would suggest that impaired regulation of level of goal/action identification may potentially be involved in the onset and maintenance of psychopathology. This section summarizes evidence that level of goal/action identification causally influences a range of symptoms and processes involved in psychological disorders. This is also further evidence consistent with the hypothesis that different levels of goal/action identifications have distinct functional effects. A conservative criterion for inclusion was adopted with reference to evidence reviewed: for inclusion in the current paper, each study needed to a) involve a manipulation or measure of level of goal/action identification; b) involve a dependent variable that was relevant to well-validated symptoms and processes involved in psychological disorders, such as emotional response, repetitive thought, and self-control.

Before reviewing each of these symptoms and processes in turn, the paper will briefly consider one further relevant literature, which suggests that level of goal/action identification is functionally associated with psychological distance and visual perspective, because these findings indicate that evidence concerning psychological distance and visual perspective may also be relevant to this review.

### 4.1 Level of goal/action identification and psychological distance

In addition to its primary derivation from control theory accounts, this analysis also draws on recent research conducted within the framework of Construal Level Theory (CLT; see reviews in Trope & Liberman, 2003; Trope, Liberman & Wakslak, 2007), which examines the effects of psychological distance on the construal of events and behaviors. CLT argues that there is a strong functional connection between psychological distance and the abstractness of representations of behaviors, events, and objects. Psychological distance is defined as the distance of an object or event in space, time, social distance, or likelihood from the directly experienced self in the here-and-now. CLT proposes that the greater the perceived psychological distance, the more likely an object or event will be construed in an abstract way (and vice versa). Thus, experiencing sensory details in the present moment would be psychologically close (and correspondingly afford concrete construal), whereas imagining an unlikely event occurring to another in several years would be psychologically distant (and afford more abstract construal).

This relationship between psychological distance and level of construal is proposed to emerge from individuals learning implicitly over time that distance typically reduces the availability of information necessary for concrete construal, whilst retaining information relevant for abstract information, such that it is more adaptive to construe distal events more abstractly. For example, “my birthday tomorrow” has considerable contextual detail concerning the likely environment and people present, whilst “my birthday in 10 years” lacks such contextual detail. As such, it would be more functional to adopt abstract construals for distant events (parallel arguments apply for objects and events at greater spatial distance or less likelihood). CLT argues that this tendency to construe more distal events and objects more abstractly is overlearnt and becomes a generalized heuristic, such that distance and level of construal are automatically associated, each bidirectionally causing the other. Consistent with this hypothesis, there is evidence that manipulating psychological distance causally influences the level of abstraction at which behaviors and events are represented, and vice versa, demonstrating the functional connection and equivalence between distance and level of abstraction (see Trope et al., 2007 for review). For the purposes of the current analysis, although construal level theory has a broad definition of abstraction that incorporates multiple dimensions, some of which may be unrelated (see Fujita et al., 2006, including means versus ends, primary versus secondary features, pro versus cons, alignable versus non-alignable features, pictorial versus linguistic), “consistent with action identification theory (Vallacher & Wegner, 1987), the representation of action in terms of means–ends relationships is viewed as an important dimension of level of construal of instrumental actions” (Fujita et al., 2006, p. 352). Moreover, within control theory, as noted earlier, different levels of identification can also be applied to objects, events, and outcomes, as a function of the generation of reference values and perceptual signals at different levels within the hierarchy. Importantly, the data generated within the study of construal level theory has directly shown that psychological distance can influence the prepotent level of goal/action identification adopted. For example, actions were construed in abstract *why* terms rather than concrete *how* terms when they pertained to the more distant future (Liberman and Trope, 1998), to greater spatial distance (Fujita et al., 2006), and to lower probability events (Wakslak, Trope, Liberman, & Alony, 2006). Moreover, the reverse relationship has been shown, with abstract *why* levels of action identification leading to estimates that the action would occur more distantly in time and space and be less likely to occur than concrete *how* levels of action identification (Liberman, Trope, Macrae, & Sherman, 2007; Wakslak & Trope, 2009), demonstrating the functional connection and equivalence between distance and level-of-identification.^3^

This link with psychological distance suggests that the most concrete level of identification will tend to be focused on direct and immediate experience in the present moment (e.g., when driving to a friends to return study notes, a highly concrete level of identification would focus on the sensation of gripping the steering wheel). In contrast, a more abstract level of identification will tend to be more focused on underlying ends, meanings, and consequences with reference to principles (e.g., “follow through on commitments”, which is relatively decontextualized as it can be applied in different ways at different times and places) or to system concepts (e.g., “to be responsible”, which necessarily applies across a time frame extended beyond the present moment). As such, a more concrete level of goal/action identification will tend to be more present-focused than a more abstract level of goal/action identification. Of course, as noted earlier, when there is extensive procedural knowledge specifying the links between the abstract purpose and the concrete means necessary to achieve it, such as when an activity is well-practised or straightforward, even abstract levels in the hierarchy will be present-focused through their engagement with the lower level control systems that interface with the present environment. For example, Carver and Scheier (1990, p. 21) provide the example of an individual “who is presently attempting to conform to his ideal self-image, by using the principle of kindness to guide his actions, a principle that presently is being manifest through the program of shovelling snow from a neighbor's sidewalk”, a program that guides the reference values for sequences such as “lifting the shovel”, which in turn informs the reference values for the sensations of gripping, etc. Thus, within the current analysis, the adoption of a prepotent operational level of goal/action identification that is abstract will tend to be associated with distance from present-focused, here-and-now direct experience, but only in the context of a dissociation between higher levels and lower levels in the control hierarchy, that is, in unfamiliar, complex, difficult or stressful situations, where there is not well-developed procedural knowledge linking abstract ends to concrete means. Thus, level of goal/action identification is related to the degree of direct contact with experience (concrete) versus separation and distance from direct experience (abstract), under the unfamiliar and stressful circumstances that are highly relevant to psychopathology. Contact with experience is increasingly viewed as clinically important: (a) experiential avoidance, defined as attempts to avoid internal experiences (e.g., thoughts and feelings) is implicated in the maintenance of a range of disorders (Hayes, Wilson, Gifford, Follette & Strosahl, 1996); (b) treatments that emphasise immediate here-and-now experiencing have beneficial effects on psychopathology (e.g., mindfulness-based CBT, Teasdale et al., 2000; Kuyken et al., 2008).

Related to the concept of psychological distance, Libby, Shaeffer and Eibach (2009) demonstrated that level of goal/action identification is also functionally connected with visual perspective (or vantage point). Libby et al. (2009) found that an observer perspective (third-person perspective, “seeing oneself in a scene”) is functionally equivalent to an abstract *why* level of identification, whereas a field perspective (first person perspective, “seeing through one's own eyes”) is functionally equivalent to a concrete *how* level of goal/action identification. Experimentally manipulating visual perspective (field versus observer) influenced the level of goal/action identification used to describe an imagined or perceived object, and vice versa, indicating a bidirectional causal relationship between visual perspective and level of goal/action identification. This is further evidence that level of goal/action identification may be associated with psychological disorder, since visual perspective of personal memories and images is implicated in psychopathology (e.g., Kuyken & Moulds, 2009; Spurr & Stopa, 2003).

### 4.2 Emotional response

Disturbed affect, such as increased depressed mood, increased anxiety, or reduced experience of pleasure (anhedonia), is a key symptom in mood and anxiety disorders. Thus, evidence that level of goal/action identification can influence emotional response to situations would suggest that (dys) regulation of level of goal/action identification could potentially be involved in these disorders. There is evidence that level of goal/action identification influences the emotional response to stressful and negative situations. First, experimentally manipulating level of goal/action identification influences emotional response to a subsequent anagram stress failure task (Moberly & Watkins, 2006; Watkins et al., 2008). Level of goal/action identification was manipulated by asking participants to imagine emotional scenarios, with instructions inducing either concrete (“Focus on *how* this event happened, and imagine in your mind as vividly and concretely as possible a ‘movie’ of how this event unfolded”) or abstract identifications (“Think about *why* this event happened, and analyze the causes, meanings and implications of this event”). Moberly and Watkins (2006) found that high ruminators trained to adopt an abstract level of processing showed greater reduction in positive affect following the failure than high ruminators trained to adopt a concrete level of processing or low ruminators in either condition. Watkins et al. (2008) found that participants trained to adopt a concrete level of processing demonstrated smaller reductions in positive affect and smaller increases in negative affect to the subsequent failure than participants trained to adopt an abstract level of processing.

Second, Watkins (2004) randomly allocated participants to expressive writing about a previously induced failure in either an abstract way (e.g., “*Why* did you feel this way?”) or a concrete way (e.g., “*How* did you feel moment-by-moment?”). Level of goal/action identification influenced emotional recovery from the failure: At higher levels of trait rumination, levels of negative mood 12 h after the failure were greater, but only in individuals who wrote abstractly and not in individuals who wrote concretely. Third, Houser-Marko and Sheldon (2008) examined the effect of framing failure or success feedback on a verbal skills task either abstractly (higher-order ends) or concretely (means to achieve those ends). Participants who framed failure abstractly reported significantly greater negative affect, significantly lower positive affect, and significantly less positive expectancy for future performance than participants who framed failure concretely or participants who received success feedback. In a follow-up naturalistic study, when progress on a personal goal was absent, participants allocated to process progress at an abstract level experienced greater decreases in mood and expectancy than participants who construed progress concretely.

Fourth, voluntarily recalling an emotional event in specific detail produces less emotional response than recalling it at a more general level (Neumann & Philippot, 2007; Philippot, Baeyens & Douilliez, 2006; Philippot, Schaefer & Herbette, 2003) and practice at recalling specific, contextualized autobiographical memories reduces the negative experience to a subsequent stressful task relative to practice at recalling general, decontextualized memories (Raes, Hermans, Williams & Eelen, 2006). Fifth, the effect of visual perspective adopted when recalling an experimentally induced experience of exclusion was found to causally influence the negative effects of ostracism, with negative effects only persisting for participants allocated to an observer perspective (Lau, Moulds & Richardson, 2009). The field perspective, functionally connected with a concrete level of goal/action identification (Libby et al., 2009), resulted in faster recovery from a negative event.

There is less evidence concerning the effects of level of goal/action identification on response to positive events. People with low self-esteem (LSE) have a tendency to think concretely about recent compliments, whereas people with high self-esteem tend to think abstractly about recent compliments, focusing on their general meanings and implications (Marigold, Holmes & Ross, 2007). Moreover, when people with LSE are induced to think abstractly about a recent compliment from a romantic partner, they report greater state self-esteem and greater security in their relationship than LSE people induced to think concretely about a recent compliment. Thus, there is preliminary evidence that being abstract may enhance the emotional effects of positive events, and that this tendency is reduced in LSE people, who are more vulnerable to mood/anxiety disorders.

In summary, there is consistent evidence that when faced with failure or stress, adopting a concrete level of goal/action identification results in less increase in negative affect and/or less reduction in positive affect than adopting an abstract level of goal/action identification. This is further evidence consistent with control theory and AIT hypotheses that it may be adaptive to shift to a concrete level of identification when faced with difficulties or stress, and suggests that the tendency to become more concrete to negative outcomes may be functional. Moreover, these findings suggest that level of goal/action identification causally influences emotional response, indicating that level of goal/action identification could potentially contribute to disturbed affect in mood and anxiety disorders. These studies did not use clinical samples, so we do not know whether these effects would generalize to patient groups. However, a clinical intervention focused on training individuals to adopt more a more concrete processing style in response to difficulties reduced symptoms of depression, relative to a no-training control, in dysphoric individuals, of whom 50% met criteria for a current episode of major depression (Watkins, Baeyens & Read, 2009).

### 4.3 Repetitive thought

Repetitive thought (RT) focused on negative content, such as depressive rumination and worry, is a common concomitant of mood and anxiety disorders, and has been implicated as an important process in the onset and maintenance of both depression and anxiety (for full review see Watkins, 2008). Moreover, RT about negative content has been identified as a definite transdiagnostic process (Ehring & Watkins, 2008; Harvey et al., 2004; Watkins, 2009a). Prospective longitudinal studies have found that depressive rumination predicts the onset and maintenance of episodes of major depression, as well as symptoms of depression (e.g., Nolen-Hoeksema, 2000; Spasojević & Alloy, 2001). Similarly, chronic worry is a central and defining characteristic of Generalized Anxiety Disorder, and post-event rumination is a typical process in social anxiety. Rumination about trauma predicts the persistence of post-traumatic stress disorder in prospective longitudinal studies from 6 months to 3 years later (e.g., Ehlers, Mayou & Bryant, 1998, 2003). Thus, RT is an important pathological process across psychological disorders. As such, any data implicating level of goal/action identification in pathological RT would be strong evidence that level of goal/action identification may have transdiagnostic effects.

Critically, a recent integrative review (Watkins, 2008) concluded that RT with negative consequences, such as found in mood and anxiety disorders, is characterized by (a) a focus on negative content (e.g., problems and symptoms) coupled with (b) a processing style characterized by an abstract level of goal/action identification focused on meanings and implications, and asking “why?”. This conclusion was based on evidence that: (i) unhelpful RT is characterized by an abstract level of processing and (ii) manipulating level of goal/action identification influenced the consequences of RT (see Watkins, 2008). As an example of (i), depressive rumination is characterized by an abstract level of identification since it is focused on meanings, consequences, implications, and “why” questions, and involves reduced concreteness of thinking (Watkins & Moulds, 2005a, 2007). As an example of (ii), in depressed patients, compared to abstract rumination focused on meanings and implications, concrete rumination focused on direct experience reduced negative global self-judgments (Rimes & Watkins, 2005), improved social problem solving (Watkins & Moulds, 2005a), and increased specificity of autobiographical memory recall (Watkins & Teasdale, 2001, 2004). Since these processes are implicated in the onset and maintenance of depression, these findings suggest that concrete rumination reduces cognitive processing implicated in increased vulnerability for depression, relative to abstract rumination.

Similarly, in an analogue study of post-traumatic stress symptoms, undergraduates watched a distressing film showing the aftermath of motor vehicle accidents, known to induce negative affect and intrusions, and were then randomly allocated to abstract rumination, concrete rumination, or distraction (Ehring, Szeimies & Schaffrick, 2009). Across time, abstract rumination resulted in slower recovery of negative affect than concrete rumination or distraction. Moreover, concrete rumination resulted in fewer negative intrusions than abstract rumination and distraction, which did not differ from each other. Thus, these results suggest that abstract rumination may be particularly unconstructive following exposure to a distressing event.

Thus, there is evidence that level of goal/action identification influences whether RT has constructive or unconstructive consequences and whether it becomes pathological. Since negative RT is an important psychopathological process judged to be transdiagnostic, this suggests that level of goal/action identification and its regulation may be important across a range of psychological disorders.

### 4.4 Personal problem solving and planning

Difficulties in problem solving and poor planning are characteristic of many psychological disorders. Both the reduced concreteness theory (Stöber & Borkovec, 2002) and the AIT (Vallacher & Wegner, 1987) hypothesize that a more concrete level of goal/action identification provides more elaborated and contextual detail about the specific means, alternatives, and actions by which to best proceed when faced with difficult, novel or complex situations. Similarly, Leary, Adams and Tate (2006) argued that abstract construal about the evaluative or interpersonal implications of one's behavior interrupts the smooth performance of behaviors, whereas, more concrete construal benefits task performance by (a) focusing attention on the immediate demands of the present situation, (b) reducing anxiety, and (c) requiring less effort and using up less self-regulatory resources. For example, a soccer player would perform better when focusing on how to take the penalty, rather than when thinking about the implications of not scoring. Consistent with these hypotheses, a concrete level of goal/action identification is associated with better problem solving (Watkins & Baracaia, 2002; Watkins & Moulds, 2005a). For example, prompting an abstract level of goal/action identification (questions like “Why did this problem happen?”) impaired social problem solving in a recovered depressed group, who performed as well as never-depressed participants in a no-prompt control condition, whereas prompting a concrete level of goal/action identification (questions like “How are you deciding what to do next?”) ameliorated the problem-solving deficit normally found in a group of currently depressed patients (Watkins & Baracaia, 2002). Moreover, the use of a concrete level of goal/action identification reduces anxiety and/or improves task performance, especially when the task is considered difficult or occurs under conditions of high cognitive load (e.g., Gollwitzer & Sheeran, 2006; Vallacher et al., 1989; Webb & Sheeran, 2003).

Furthermore, there is evidence that level of goal/action identification during mental simulations influences the effectiveness of planning (Taylor, Pham, Rivkin & Armor, 1998). Students who imagined the process of *how* to take steps towards obtaining a high exam grade studied more and obtained better grades than students who imagined the outcome of obtaining a high grade (i.e., focus on ends, an abstract level of goal/action identification) or students who monitored their studying with no mental simulation (Pham & Taylor, 1999; Taylor et al., 1998). This effect of process simulation versus outcome simulation on exam performance was mediated by a reduction in anxiety and by increases in planning. Repeated imagining of an ongoing stressful event, how it happened, and its associated emotions produced more positive affect and greater report of active coping after 1 week than imagining having resolved the situation or not imagining the event at all (Rivkin & Taylor, 1999).

Thus, consistent with control theory and AIT, there is evidence that adopting a more concrete level of goal/action identification is adaptive to cope with personally relevant difficulties and to solve interpersonal problems. Again, these findings suggest that the tendency for healthy controls to become concrete in response to negative mood is adaptive, suggesting that individuals effectively regulate their level of goal/action identification. However, being impaired in the ability to become concrete to difficulties may be a risk factor for psychological disorders.

### 4.5 Self-regulation, inhibition, and impulsivity

Poor self-regulation and impulsive responses are characteristic of a number of psychological disorders including eating disorders, alcohol and substance abuse disorders, manic episodes within bipolar disorder, impulse-control disorders, and personality disorders. Thus, if level of goal/action identification causally influences self-regulation and impulsivity, (dys)regulation of level of goal/action identification may be a factor relevant to the onset and/or maintenance of these disorders. As noted earlier, control theory and AIT predict that an abstract level of goal/action identification will increase self-control and reduce failures of self-regulation. These theories propose that level of goal/action identification will influence self-regulation in situations of self-control conflict that involve resisting temptation or ignoring distraction, because in these situations there is a conflict between benefits at the more abstract level (e.g., long-term gain) versus costs at the concrete level (e.g., short-term loss or pain). For example, an individual with the abstract goal of “losing weight”, when faced with a delicious-looking piece of cake, is more likely to resist temptation if he adopts an abstract level of identification focused on long-term goals than a concrete level of identification focused on the immediate sensory experience. Similarly, Baumeister and Heatherton (1996) proposed that one proximal cause of self-regulation failure is the failure to focus awareness beyond the immediate stimuli, that is, to fail to transcend the immediate situation by seeing it in the context of more distal, abstract concerns. Consistent with this, Karniol and Miller (1983) demonstrated that failure to delay gratification was often preceded by shifts in attention to the immediate reward. Baumeister and Heatherton (1996) proposed that some impulsive behavior (e.g., aggression) could be understand in terms of acting in response to short-term concerns whilst failing to consider long-range implications, leading to acting in “the heat of the moment”. They further hypothesized that a “great deal of binge behavior, whether it be shopping, gambling, eating, drinking, or having sex, seems to result when people are seeking to keep their attention focused on immediate, concrete stimuli as a means of keeping it away from some threatening or upsetting thoughts” (Baumeister & Heatherton, 1996, p. 5). Thus, there is considerable theoretical rationale for level of goal/action identification influencing self-regulation in clinical contexts, with a concrete level-of-identification implicated in poorer resistance to temptation. Remember that within control theory accounts, only the levels subordinate to the currently prepotent operational level of goal/action identification will be engaged — that is, for an individual who is momentarily processing goals and events at a relatively concrete level, there is little or no reference to higher-order goals, and thus, the individual could easily act at odds to abstract goals (and effectively during the time a more concrete level is prepotent, higher-order goals are temporarily disconnected from the present moment).

Consistent with these theories, a more abstract level of goal/action identification is associated with less impulsivity and greater self-control. First, a habitual tendency towards a more abstract level of goal/action identification was associated with a greater self-reported tendency to persevere on a course of action, less self-reported impulsiveness, and greater temporal stability in behavior (Vallacher & Wegner, 1989). Moreover, in a sample of juvenile detainees, individuals who tended to adopt a more abstract level of identification were less likely to have an offense record and less likely to experience school trouble, suggesting an ability to forestall some negative consequences of their behavior (Vallacher & Wegner, 1989). Second, eating binges seem to be characterized by an immersion in immediate sensation (an extremely concrete level of identification) and a cessation in monitoring the meanings and purpose of the behavior (i.e., disengagement from more abstract levels of identification, Heatherton & Baumeister, 1991). Third, impulsive and risky behavior is associated with the narrow focus on the here-and-now that is characteristic of a more concrete level of identification. The tendency to adopt a present time-perspective, in which immediate here-and-now stimuli are the primary focus in determining actions, was highly related to risky driving (e.g., speeding and drinking under the influence of alcohol, Zimbardo, Keough & Boyd, 1997), to more frequent smoking, consumption of alcohol, and drug use (Keough, Zimbardo & Boyd, 1999), and to more frequently missing submission deadlines for academic work (Zimbardo & Boyd, 1999). Although these behaviors do not necessarily always reflect a failure of self-control and could reflect the enactment of desired actions (albeit whilst minimising their potential detrimental long-term effects), a significant proportion of drinking, drug use, risky behavior, etc., probably does reflect impulsive and contextually-driven actions at odds with abstract personal goals. However these findings are limited in that the measures are self-report and the findings are correlational, leaving the causal direction between level of goal/action identification and poor self-regulation unresolved.

However, a number of experimental studies have shown that manipulating level of goal/action identification causally influences self-control on a range of experimental tasks, with more abstract level of identification producing greater resistance to temptation than more concrete level of identification (Fujita & Han, 2009; Fujita et al., 2006; Schmeichel & Vohs, 2009). For example, relative to participants primed into a concrete level of goal/action identification by questions asking “how?” they would enact particular goals, participants primed into an abstract level of goal/action identification by questions asking “why*?*” they would adopt particular goals, demonstrated decreased preferences for immediate over delayed outcomes, greater physical endurance (holding a handgrip for significantly longer) (Fujita et al., 2006), and associated temptation (“candy bars”) with negativity on an implicit association test (Fujita & Han, 2009). Similarly, describing situations that involve self-control conflict (e.g., eating a piece of cake when on a diet) in more abstract terms resulted in more negative evaluations of temptations than when situations were described in concrete terms (Fujita et al., 2006). Schmeichel and Vohs (2009) demonstrated that affirming the self at an abstract level (writing *why* one pursues a particular personal value) resulted in more delay of gratification on a computer game than affirming the self at a concrete level (writing *how* one pursues personal values), and counteracted depletion of self-regulatory resources by prior engagement in a self-control task. Since depleting self-regulatory resources by prior engagement in a self-control task causes more concrete level of identification (Vohs & Schmeichel, 2003), increases alcohol consumption (Muraven, Collins & Neinhaus, 2002), and leads to loss of self-control in eating (e.g., eating more unhealthy food, Vohs & Heatherton, 2000), a more abstract level of identification may counteract such losses of self-control.

Thus, level of goal/action identification is causally implicated in inhibiting prepotent and impulsive responses (such as resisting temptation and delaying gratification) consistent with the theoretical models described earlier. Since difficulties in inhibiting prepotent responses are characteristic of various psychological disorders (e.g., binge eating), and impulsivity is among the strongest prospective predictors of later addictive behaviors (de Wit, 2009), these findings suggest that (dys)regulation of level of goal/action identification may influence symptoms in these disorders.

### 4.6 Procrastination, initiation of goal pursuit, and self-motivation

Difficulties in initiating goal pursuit is an important symptom across those psychological disorders characterized by reduced inactivity and avoidance such as major depression, for which loss of motivation and difficulties making decisions are diagnostic criteria. A number of theoretical perspectives suggest that a more concrete level of identification may be adaptive in initiating goal pursuit (in the context when there is little prior procedural specification from abstract goals to the programs and sequences of behaviors necessary to achieve the goal, such as when the goal pursuit is not well established or well-practised). As noted earlier, setting more concrete, specific, and attainable sub-goals may facilitate the delivery of action by providing immediate incentives and guides for performance (Bandura & Schunk, 1981) and by reducing the delay of rewards, whereas distal goals may be too far removed in time to effectively mobilize effort or to direct what one does in the here-and-now. Moreover, a concrete level of identification that specifies when, where, and how an action will occur may facilitate goal pursuit and initiation of action by transferring control of the behavior to cues in the situational context rather than relying on individual volition (Gollwitzer, 1999). This is conceptually equivalent to the argument re impulsivity: adopting a concrete level of goal/action identification as the prepotent operational level transfers more control of a behavior to contextual cues, such that less volitional effort/executive resources are required to initiate it (or more effort required to inhibit it if at odds with higher-order goals).

Consistent with these hypotheses, adopting a concrete level of goal/action identification can reduce procrastination and hastens the initiation and execution of behaviors. First, forming concrete plans concerning the when, where, and how of pursuing a goal leads to earlier enactment of goal-directed behavior, quicker initiation of responses, and better goal completion (Gollwitzer & Sheeran, 2006; Webb & Sheeran, 2003). Second, McCrea, Liberman, Trope and Sherman (2008) found that manipulating level of goal/action identification causally influenced extent of procrastination. In three studies, participants were asked to respond to a questionnaire via email within 3 weeks. In each study, the questionnaire was designed to induce either an abstract or a concrete level of processing. In all three studies, participants in the concrete condition returned the questionnaire by email more quickly than participants in the abstract condition. Third, Bandura and Schunk (1981) found that for children who had difficulties in mathematical tasks, a program of self-directed learning was more effective when participants were provided with proximal goals, relative to children assigned distal goals or no goals at all. In the proximal subgoal condition, children progressed rapidly in self-directed learning, achieved substantial mastery of mathematical operations, showed greater task persistence, and developed a sense of personal efficacy and intrinsic interest in mathematical activities, whereas distal goals had little effect. Similarly, Stock and Cervone (1990) found that the assignment of a proximal subgoal (relative to no subgoal) on a complex problem-solving task increased initial strength of self-efficacy for completing the problem, which in turn led to greater persistence on the task.

## 5 Evidence that level of goal/action identification is abnormal in psychological disorders

The previous section provides evidence that level of goal/action identification can causally influence symptoms and processes associated with psychological disorders, with the implication that dysregulation of level of goal/action identification could contribute to psychopathology. The evidence presented is broadly supportive of the distinctions predicted in Table 1. However, because the majority of these studies used non-clinical samples (see exceptions in patients with major depression, Watkins & Baracaia, 2002; Watkins & Moulds, 2005a; Watkins & Teasdale, 2001, 2004), there still remains the question of whether level of goal/action identification is actually dysregulated in psychological disorders. There are two types of evidence for dysregulation of level of goal/action identification. First, there may be evidence that there is an asymmetrical bias in the level of goal/action identification adopted in a psychological disorder relative to controls. For example, the finding that individuals with a particular psychological disorder tend to adopt a more abstract level of identification for negative outcomes and events than controls would indicate that level of goal/action identification is abnormal and probably dysfunctional in that disorder, since the level of goal/action identification adopted would be at odds with the level of goal/action identification found to be most adaptive. Moreover, any asymmetrical bias towards one level of goal/action identification implies reduced responsiveness to situational context and impaired regulation, e.g., a bias towards abstract identification of negative events suggests that level of goal/action identification does not become more concrete in response to difficulty. Second, there may be direct evidence of such impaired regulation of level of goal/action identification, which requires the demonstration that level of goal/action identification does not shift with changing circumstances (e.g., mood inductions) in the functional way that is typically observed in healthy controls. This section summarizes both types of evidence across a selected range of psychological disorders.

### 5.1 Major depression

There is extensive evidence that the level of goal/action identification adopted in major depression is abnormal and dysfunctional, with patients with depression tending to adopt more abstract levels of goal/action identification, at least for negative information, than non-depressed controls. First, relative to non-depressed individuals, currently depressed and formerly depressed individuals tend to overgeneralize from a single negative event, such that a single failure is represented in terms of a global and characterological personal inadequacy (e.g., “I am worthless”, an abstract level of identification at the self-concept level), rather than in terms of specific behavior (a concrete level of identification) (e.g., Carver & Ganellen, 1983; Carver, La Voie, Kuhl, & Ganellen, 1988; Eisner, Johnson & Carver, 2008; Ganellen, 1988; Wenzlaff & Grozier, 1988). Negative generalization prospectively predicts increases in depressive symptoms in conjunction with negative events (Carver, 1998) and mediates the effects of failure on emotional reactivity (Brown & Dutton, 1995; Kernis, Brockner & Frankel, 1989). Thus, an abstract level of identification is implicated in the maintenance of symptoms.

Second, as noted earlier, depressive rumination is characterized by an abstract level of goal/action identification (Watkins, 2008). Such depressive rumination maintains over general autobiographical memory recall and global negative self-evaluations, relative to distraction or more concrete self-focus (Rimes & Watkins, 2005; Watkins & Teasdale, 2001, 2004). Third, there is evidence of reduced concreteness of thinking in depressed patients when thinking about problems (Cribb, Moulds & Carter, 2006; Watkins & Moulds, 2007). Fourth, Emmons (1992) found that individual differences in preferred level of goal/action identification for personal strivings (important recurring goals) and spontaneous thoughts were associated with psychological well-being, with more abstract thoughts and more abstract personal strivings associated with greater negative affect assessed over 21 days using an experience sampling methodology and with more depressed symptoms.

Fifth, studies of visual perspective in depression also suggest that depression involves a bias towards an abstract level of goal/action identification. Depressed adults (Lemogne et al., 2006), and formerly depressed patients (Bergouignan et al., 2008; Kuyken & Moulds, 2009) are characterized by a tendency to retrieve a greater proportion of observer memories than non-depressed individuals. Given evidence that visual perspective is functionally equivalent to level of goal/action identification (Libby et al., 2009), this tendency towards an observer perspective in major depression is further evidence that depressed patients tend towards a more abstract level of goal/action identification for personally relevant information. Moreover, adoption of an observer perspective seems to be associated with attempts to evaluate oneself and with depressive rumination (Kuyken & Moulds, 2009; Libby, Eibach & Gilovich, 2005; Williams & Moulds, 2007), consistent with an abstract level of identification. Kuyken and Moulds (2009) found that vantage perspective was a moderator of treatment response to mindfulness-based cognitive therapy: participants who reported more field perspective memories pre-treatment had lower levels of post-treatment depression, controlling for baseline levels of depression.

Thus, there is extensive evidence indicating that patients with major depression tend to adopt a more abstract level of goal/action identification, which is at odds with the hypothesized and observed functional linkage between sad affect and problems and a concrete level of goal/action identification found in non-depressed participants. Moreover, there is both experimental evidence (e.g., Watkins & Baracaia, 2002; Watkins & Moulds, 2005a) and longitudinal prospective evidence that a concrete level of goal/action identification (Carver, 1998; Kuyken & Moulds, 2009; Williams et al., 2007) leads to more adaptive consequences in depressed patients, suggesting that: (a) abstract level of goal/action identification may contribute to depression, rather than simply be a consequence of depression; (b) there is tendency for an abstract level of goal/action identification to become prepotent despite being maladaptive.

Based on this evidence, Watkins (2008) hypothesized that currently depressed individuals are impaired in flexibly regulating level of goal/action identification in response to situational demands, and consequently are less likely to use concrete representations in response to increases in sad mood. There is preliminary evidence consistent with this *dysregulation hypothesis*. First, when asked to generate counterfactual thoughts about a negative event, individuals with mild-to-moderate depressive symptoms generated a more concrete level of goal/action identification focused on specific, controllable behaviors, relative to non-depressed individuals and severely depressed individuals, whereas severely depressed individuals generated more a abstract level of goal/action identification focused on uncontrollable, global, enduring qualities of the self than non-depressed and mildly depressed individuals (Markman & Miller, 2006). Thus, mild depressive symptoms are associated with the adaptive regulation of level of goal/action identification in response to mood, but more extreme depressive symptoms are associated with dysregulation of this process.

Second, Watkins, Moberly, and Moulds (submitted for publication) directly tested this hypothesis by measuring level of goal/action identification on the Behavioral Identification Form after happy and sad mood inductions in never-depressed controls and currently depressed patients. Consistent with Watkins (2008) hypothesis, increases in sad mood were associated with shifts towards more a more concrete level of goal/action identification in never-depressed individuals, but not in depressed patients. These findings suggest that the proposed functional association consistently found between sad mood and level of goal/action identification is impaired in major depression.

Third, Takano and Tanno (2010) used an experience sampling method to investigate the relationship between self-focus, concreteness of thinking, depressive symptoms and concurrent negative affect in daily life. Thirty-one undergraduates recorded their negative affect, ruminative self-focus and concreteness of thinking eight times a day for 1 week. Multilevel modeling found that individuals with increasing levels of depressive symptoms engaged in more abstract thinking in daily life. Furthermore, there was a significant negative correlation between intra-individual standard deviations of concreteness and depression, suggesting that concreteness fluctuates less as levels of depression increases, which the authors interpreted as indicating that “individuals with increasing levels of depression might have a malfunction in switching their levels of concreteness” (Takano & Tanno, 2010, p.424). Moreover, consistent with the hypothesis that level of goal/action identification may influence the negative consequences of repetitive thought, ruminative self-focus was significantly positively associated with negative affect but only when associated with abstract thinking.

In summary, major depression is associated with a bias towards adopting a more abstract level of goal/action identification, especially in the context of negative affect and limited goal progress, even when this is unhelpful. This pattern is consistent with and helps to explain the wider symptomatology of depression such as overgeneralization, rumination, procrastination, and reduced activity. Moreover, there is preliminary evidence that this bias may reflect dysregulation of level of goal/action identification in response to changes in mood. However, to date, the level of goal/action identification adopted in response to positive information has not been evaluated — the findings of Marigold et al. (2007) that LSE individuals tend to identify positive events (compliments) concretely suggests that patients with major depression may act similarly.

### 5.2 Bipolar disorder and mania

When considering level of goal/action identification within bipolar disorder, it is important to distinguish between the level of goal/action identification adopted in an episode of bipolar depression, an episode of mania, or during a period of euthymic mood and, where possible, to control for each set of symptoms. The majority of studies have tended to examine participants in an euthymic state and thus make it hard to assess what the level of goal/action identification would be within a depressed or manic episode.

The theories and evidence already reviewed suggest various (potentially complementary) hypotheses concerning the level of goal/action identification adopted in bipolar disorder. First, it is plausible that bipolar depression would show a similar level of goal/action identification to unipolar depression, i.e., a tendency to be more abstract in response to negative outcomes. The extant evidence is consistent with this hypothesis, since the level of current depressive symptoms in individuals with bipolar disorder is correlated with measures of more abstract levels of identification such as negative overgeneralization and depressive rumination (Eisner et al., 2008; Johnson, McKenzie & McMurrich, 2008).

Second, given that distractability, impulsivity, and engaging in high risk behaviors are diagnostic criteria for mania, it could be hypothesized that mania is characterized by the adoption of a concrete level of goal/action identification in response to appetitive and rewarding stimuli, resulting in less self-control under these circumstances (Vallacher & Wegner, 1989; Zimbardo et al., 1997). Such an analysis is consistent with the clinical impression that patients in a manic episode pay less heed to the long-term consequences of their actions. However, to date, the level of goal/action identification during a manic or hypomanic episode has not been assessed, nor has the level of identification typically adopted in response to appetitive stimuli.

Third, it is a plausible hypothesis that vulnerability for mania is characterized by a propensity towards exaggerated abstract level of goal/action identification of positive outcomes, leading to elevated positive mood and grandiosity (i.e., more extreme version of high self-esteem individuals seen in Marigold et al., 2007). Consistent with this hypothesis, in an undergraduate sample, increased risk for development of bipolar disorder, as indexed by elevated scores on the Hypomanic Personality Scale, was significantly associated with positive generalization, defined as the tendency to generalize from a good experience in one domain of life to broader aspects of life (Eisner et al., 2008; Carver & Johnson, 2009). Similarly, individuals with elevated hypomania scores and individuals with the diagnosis of bipolar disorder reported an elevated tendency towards positive rumination in response to positive affect relative to individuals with the diagnosis of major depression or healthy controls (Carver & Johnson, 2009; Johnson et al., 2008).

Thus, there is preliminary evidence that vulnerability for mania is associated with a more abstract level of goal/action identification for positive outcomes, whereas bipolar depression is associated with a more abstract level of goal/action identification for negative outcomes. However, not all this data was collected in a diagnosed sample, nor was the effect of mood state (i.e., in depressed or manic episode) examined. Given the literature reviewed earlier, one would expect level of goal/action identification to change with varying mood state — as such, it will be critical to assess level of goal/action identification across mania, depression, and euthymia in patients with bipolar disorder, to determine how it dynamically changes with circumstances.

### 5.3 Generalized anxiety disorder (GAD)

Since pathological worry is a primary and defining symptom of GAD, the evidence reviewed by Watkins (2008) that pathological RT is characterized by an abstract level of goal/action identification suggests that GAD is also characterized by a predisposition towards adopting an abstract level of goal/action identification as the prepotent operational level, for negative events. A similar prediction is made by the reduced concreteness theory, which proposes that worry is predominantly experienced in a more abstract-verbal form rather than in a more concrete-visual imagery form, and that this reduced concreteness leads to negative consequences for problem solving and affect regulation (Borkovec et al., 1998; Stöber, 1998). Consistent with this theory, worry is predominantly experienced in a verbal form rather than in images (Borkovec et al., 1998; Borkovec, Robinson, Pruzinsky & Depree, 1983). Moreover, elaborations of problems about which participants worry are independently and blindly rated as more abstract and less concrete than problems about which participants do not worry for patients with GAD (Stöber, 1998; Stöber & Borkovec, 2002; Stöber, Tepperwien & Staak, 2000). Thus, patients with GAD demonstrate a biased tendency towards a more abstract level of processing. Given that in healthy controls, negative mood is associated with a more concrete level of goal/action identification, this is indirect evidence that level of goal/action identification is dysregulated in GAD, although there has not been any direct test of how level of goal/action identification shifts in response to changing circumstances or mood state. Moreover, experimental studies suggest that this more abstract level is associated with less emotional processing, reduced cardiovascular responses to emotional stimuli, and poor problem solving (Borkovec et al., 1998).

### 5.4 Post-traumatic stress disorder (PTSD)

There is also preliminary evidence that PTSD is associated with a bias towards abstract processing of traumatic events, based on evidence that implicates (a) abstract rumination and (b) an observer perspective in the maintenance of PTSD. First, there is evidence that abstract rumination is an important psychopathological process in PTSD (see Ehring, Frank & Ehlers, 2008; Ehring & Watkins, 2008; Watkins, 2008), which prospectively predicts PTSD symptoms. Importantly, this research identified that the element of rumination associated with PTSD severity was repeated asking of “why” and “what if” type questions — that is, an abstract level of goal/action identification focusing on the meaning, implications, and consequences of the trauma. Moreover, experimental studies suggest that abstract rumination (characterized by asking “why”?) contributes to the maintenance of symptoms in PTSD, such as duration of negative affect and number of negative intrusions, following exposure to an analogue stressor such as film footage of road traffic accidents (Ehring et al., 2008; Ehring et al., 2009).

Second, there is evidence that PTSD is characterized by a tendency towards an observer perspective when recalling autobiographical memories of the trauma. Trauma-exposed individuals with PTSD recall more observer trauma memories than those without PTSD (McIsaac & Eich, 2004). Moreover, Kenny et al. (2009) found that in a large prospective longitudinal study, initially recalling the trauma from an observer perspective was related to more severe PTSD symptoms 1 year later, and, shifting from a field to an observer perspective a year after trauma was associated with greater PTSD symptoms at 12 months. Since the observer perspective is functionally connected with an abstract level of goal/action identification, this is evidence that PTSD is characterized by a more abstract level of goal/action identification of trauma and that this abstract level of identification maintains negative affect and slows recovery.

### 5.5 Social anxiety

There is initial evidence that social anxiety is associated with a bias towards an abstract level of goal/action identification in response to anxiety-provoking events. First, post-event rumination (“post-event processing” “post-mortem thinking”) defined as “RT about subjective experiences during a recent social interaction, including self-appraisals and external evaluations of partners and other details involving the event” (Kashdan & Roberts, 2007, p. 286) has been hypothesized to contribute to the development and maintenance of social anxiety (Clark & Wells, 1995; Rapee & Heimberg, 1997). Compared to low-anxious controls, individuals with high social anxiety and patients with a diagnosis of social anxiety demonstrate significantly more post-event abstract RT following social interactions, performing mental “post-mortems” on the consequences of the interaction and evaluating their performance (e.g., Abbott & Rapee, 2004; Kocovski, Endler, Rector & Flett, 2005; Mellings & Alden, 2000). Moreover, Wong and Moulds (2009) found that following a social-evaluative task, rumination maintained anxiety in both high and low socially anxious controls relative to distraction. Furthermore, Vassilopoulos and Watkins (2009) found that in individuals high in fear of negative evaluation, a concrete rumination condition decreased ratings of the self as worthless and incompetent, pre- to post-manipulation, whereas an abstract rumination condition (thinking about the meanings and implications of current feelings) maintained such negative self-judgments, implicating abstract rumination in the maintenance of negative cognition.

Second, there is evidence that individuals with social anxiety are significantly more likely than controls to adopt an observer perspective when recalling difficult social situations (e.g., Hackmann, Clark & McManus, 2000; Wells, Clark & Ahmad, 1998; Wells & Papageorgiou, 1999). Moreover, consistent with the theories and evidence suggesting that level of goal/action identification can influence affect and performance, there is evidence that the visual perspective adopted causally influences symptoms of social anxiety (Spurr & Stopa, 2003). When high and low socially anxious participants gave speeches, adopting the observer perspective for one speech, and adopting the field perspective for the other speech, use of the observer perspective produced more frequent negative thoughts, more safety behaviors, and worse self-evaluation of performance in both groups, relative to the field perspective, as well as a strong trend towards greater anxiety (Spurr & Stopa, 2003). The observer perspective involved instructions to imagine what you look like and how you might be coming across to anyone watching (i.e., an abstract level of goal/action identification), whereas the field perspective involved instructions to be aware of details in the environment (i.e., a concrete level of goal/action identification, cf. Vallacher et al., 1987).

### 5.6 Suicide and suicidal behavior

Consistent with escape theory, there is evidence that suicidal individuals are characterized by an abstract level of goal/action identification (i.e., focused on meanings for self) and high levels of self-awareness in response to negative events, which is then followed by a concrete level of goal/action identification (i.e., focused on immediate sensation) at the point of a suicide attempt (see Baumeister, 1990). In particular, there is evidence that suicide attempters are characterized by concrete thinking that is focused on immediate movements and sensations, and guided by immediate proximal goals, rather than on more abstract implications and distal goals. Baumeister (1990) reviews evidence that genuine suicide notes tend to be relatively concrete, with a narrow immediate focus, specific instructions, and more references to concrete objects, than simulated notes.

### 5.7 Addiction: alcohol and substance abuse/dependence disorders

When considering addictive problems, such as alcohol abuse/dependence, it is important to distinguish between different patterns and functions of symptoms. Difficulties could take the form of (a) problems in resisting temptation in response to appetitive stimuli leading to substance abuse; (b) impulsive binging that is negatively reinforced as a means of avoiding distress; and (c) a consistent, habitual pattern of excessive substance use such as found in alcohol dependence. Different patterns of level of goal/action identification may relate to these different symptom patterns.

Many theories of addiction propose that addictive behaviors depend upon the relative balance between (i) automatic associations within an impulsive appetitive system such that the presence of an appetitive stimuli (whether an actual physical object or a mental representation, e.g., a craving) activates approach tendencies and (ii) reflective processing focused on the long-term consequences of such action, which controls these automatic tendencies (see Wiers, Field, & Stacy, in press). Similarly, Bickel and Marsch (2001) argued that impairments in impulsive decision making, such that behavior tends to be influenced disproportionately by its immediate consequences rather than by delayed consequences, are implicated in drug and alcohol abuse. Thus, within these theories, a currently prepotent concrete level of goal/action identification would heighten the impact of automatic context-driven associations and the impulsive system, whereas an abstract level of goal/action identification would heighten the impact of the reflective system. Consistent with this analysis, Wiers et al. (in press) predicted that focusing on the sensory (i.e., concrete) rather than on the health (i.e., abstract) aspects of an addictive behavior should activate impulsive appetitive processes. Consistent with these hypotheses, there is evidence that a concrete level of goal/action identification is associated with more frequent consumption of alcohol and increased drug use (Keough et al., 1999). Second, studies assessing “delay discounting”, which indexes the extent to which an individual chooses smaller immediate (hypothetical) rewards (e.g., money and alcohol) in preference to larger delayed (also hypothetical) rewards, find that heavy alcohol use, cocaine abuse, and heroin abuse are associated with marked discounting of delayed rewards, i.e., a preference for immediate gratification, consistent with a concrete level of goal/action identification focused on the here-and-now that has no reference to higher-order abstract goals focused on distant consequences (e.g., Kirby & Petry, 2004; Petry, 2001; Rossow, 2008; Vuchinich & Simpson, 1998). Third, there is evidence that people sometimes manage to stop severe addictive behavior after an important emotional event that makes the consequences and implications of the continued addictive behavior salient in relation to personal goals, that is, when the individual begins to identify their behavior more abstractly (Miller, 1998).

The literature on craving is also consistent with level of goal/action identification influencing impulsive appetitive behaviors. Cravings are highly intense “wishes or urges to gain pleasure, relieve discomfort, satisfy a want, or to engage in consummatory behavior associated with these outcomes”, and often involve images about the attractive features of the appetitive object or activity (Kavanagh, Andrade & May, 2005, p. 446). Craving is a reliable predictor of relapse to alcohol and drug use among addicts seeking treatment (Bottlender & Soyka, 2004; Flannery, Poole, Gallop & Volpicelli, 2003; Pahwal, Hyman & Sinha, 2008). Kavanagh et al. (2005) proposed that intense or prolonged craving results when intrusive thoughts that are activated by learned associations to internal or external antecedent events (e.g., hunger and external cues) are then further mentally elaborated through appetitive rumination. Unlike depressive rumination, such appetitive rumination is argued to typically include “the construction of sensory images that often are vivid and richly textured, lending the experience a sensory immediacy” (Kavanagh et al., 2005), that is, involving a concrete level of goal/action identification of appetitive stimuli. Consistent with this hypothesis, Kavanagh et al. (2005) reviewed evidence that concrete imagery is an important component of craving, that imagery elicits craving, and that unrelated imagery tasks reduce craving. Recently, Kavanagh, May and Andrade (2009) found that imagery of taste (which has to be experienced in a field perspective), vision and swallowing were common during craving episodes in patients receiving treatment for alcohol abuse or dependence, that more frequent imagery was associated with stronger craving, and that self-monitored daily craving frequency was associated with daily alcohol consumption.

Moreover, consistent with the escape theory account's view that substance/alcohol abuse may result from a concrete level of goal/action identification adopted as a means to escape from negative abstract self-evaluations, there is evidence linking distress and depressive rumination to increased drinking and substance use. Nolen-Hoeksema and Harrell (2002) found that high ruminators were more likely to report drinking to cope with distress and greater problematic substance use. In a prospective longitudinal sample of 496 female adolescents, rumination predicted future increases in bulimic and substance abuse symptoms, as well as onset of major depression, binge eating, and substance abuse (Nolen-Hoeksema, Stice, Wade & Bohon, 2007). In a sample of 603 community participants, distress and rumination partially mediated the relationship between self-reported childhood sexual abuse and consumptive behaviors such as increased use of food, alcohol, drugs in response to feeling upset (Sarin & Nolen-Hoeksema, 2010), suggesting that individuals adopt binge eating and drinking in order to cope with distress and the effects of rumination.

Furthermore, the effects of alcohol itself are consistent with the hypothesis that level of goal/action identification is dysregulated in addictive behavior. There is evidence that alcohol shifts individuals to a more concrete level of goal/action identification, consistent with the idea that alcohol may be used as a means to escape abstract self-evaluations (Heatherton & Baumeister, 1991). Steele and Josephs (1990, p. 923) reviewed evidence that alcohol causes “alcohol myopia” defined as “a state of short-sightedness in which superficially understood, immediate aspects of experience have a disproportionate influence on behavior and emotion”. These findings are also consistent with proposals that the acute effects of alcohol feed into the processes maintaining alcohol use (e.g., Field, Schoenmakers & Wiers, 2008), by interfering with the regulation of level of goal/action identification in a way that becomes self-perpetuating. These findings also suggest how co-morbid alcohol use may impact on dysregulation of level of goal/action identification in other disorders, leading to further impulsive behaviors (e.g., heightening the risk for suicidal attempts).

Finally, Wegner, Vallacher and Dizadji (1989) provided an AIT analysis of habitual problem drinking, such as found in alcohol dependence, which proposes that as a behavior becomes more automatic, it will be construed more abstractly such that experienced or excessive drinkers will tend to identify their drinking at an abstract level of goal/action identification in terms of its meanings (relaxation and relieving tension), whereas novice drinkers will identify their drinking at a more concrete level of goal/action identification focused on the details of the action. AIT hypothesizes that a concrete identification of a behavior provides more opportunity to monitor, change, and interrupt the behavior because it is broken down into relatively short segments of action (“lifting a glass” and “swallowing”) than an abstract identification organized in terms of global products like “relieving tension”, such that a greater number of drinks may be required before the identified action is complete in an abstract identification. Consistent with this analysis, Wegner et al. (1989) found that increasing alcohol use is associated with decreasing awareness of the details of drinking, such that inpatients with chronic alcohol problems provided more abstract descriptions of their drinking behavior than college undergraduates.

There is thus preliminary evidence consistent with abnormal level of goal/action identification in addiction, substance and alcohol disorders. However, this research still lacks (i) a fine-grained analysis of how the different levels of goal/action identification for different contexts interact (e.g., the relationship between concrete level of identification for appetitive stimuli and abstract level of identification for habitual drinking behavior — perhaps the former associated with binging and the later with dependence); (ii) close examination of the temporal sequence predicted by escape theory (negative abstract level of goal/action identification leads to concrete level of goal/action identification, to escape discrepancy at level of higher-order goals), and (iii) is limited by being mainly cross-sectional, limiting conclusions about causality.

### 5.8 Binge eating and eating disorders

Following an extensive review, Heatherton and Baumeister (1991, p. 98) concluded that “it appears that an eating binge is accompanied by focus on immediate stimuli, low-level or deconstructed thinking (i.e., short-term focus on movements and sensations) and rejection of meaningful thought (resulting in concrete, rigid, and dichotomous thinking).” Thus, the experience of an eating binge is characterized by a concrete level of goal/action identification becoming prepotent, which by function of reducing reference to higher levels in the goal system, reduces awareness of the self. Since binge eating is a key diagnostic criterion for bulimia nervosa and binge eating disorder, this indicates that a bias towards a concrete level of goal/action identification when faced with appetitive food-related cues may be present in both disorders. As noted earlier, also consistent with escape theory, abstract rumination predicts subsequent binge eating (Nolen-Hoeksema et al., 2007). There is also evidence that obese women show greater delay discounting than healthy-weight women (Weller, Cook, Avsar & Cox, 2008), consistent with a concrete level of goal/action identification for appetitive stimuli being associated with increased consumption.

## 6 Discussion

The data reviewed above suggests three lines of evidence for considering that dysregulation of level of goal/action identification may be a transdiagnostic process. First, there is evidence that different levels of goal/action identification have distinct functional consequences and that in non-clinical controls, level of goal/action identification appears to be regulated in a flexible and adaptive way to match the level of goal/action identification to circumstances. This pattern of findings suggests that regulation of level of goal/action identification is a normal and adaptive process, and, thus, a good candidate for a process that may be transdiagnostic, and that, if dysregulated, may contribute to the onset and/or maintenance of disorders. Second, there is evidence that level of goal/action identification influences symptoms and processes involved in psychological disorders. Experimental manipulations of level of goal/action identification influence emotional response to failure, the consequences of repetitive thought, delay of gratification and resistance to temptation, problem solving, and procrastination. As these outcomes are characteristic of a range of psychological disorders and causally contribute to psychopathology, this suggests that level of goal/action identification could be a variable with transdiagnostic implications. Third, there is evidence that level of goal/action identification is biased in certain psychological disorders, with evidence that there is an elevated tendency to adopt more abstract levels of goal/action identification for negative outcomes and events in depression, GAD, PTSD, and social anxiety, and evidence that addictions and binge eating involve an elevated tendency for concrete levels of goal/action identification for appetitive stimuli. The first and second lines of evidence are the most well-developed — the evidence for abnormal and dysregulated level of goal/action identification in psychological disorders is preliminary and requires further development. Nonetheless, the convergent evidence that level of goal/action identification can influence symptoms and that level of goal/action identification is abnormal in several psychological disorders relative to controls provides preliminary support for the hypothesis that dysregulation of level of goal/action identification may be a transdiagnostic process.

In sum, although these findings need to be treated with caution, they indicate that the regulation of level of goal/action identification could influence a wide range of psychological disorders and that this hypothesis is worthy of further investigation. With respect to the definitions utilised by Harvey et al. (2004) when assessing the quality of the evidence for transdiagnostic processes, these findings suggest that dysregulation of level of goal/action identification is a *possible* transdiagnostic process (defined when the majority of the evidence, which must be of at least moderate quality, indicates that the process is present in at least two disorders). There is moderate quality evidence for dysregulation of level of goal/action identification in depression, GAD, PTSD, and social anxiety. In order to confirm that dysregulation of level of goal/action identification is a definite transdiagnostic process, further research is required to examine if this dysregulation is found in all of the disorders in which it has been investigated.

Although this review has found much data that is consistent with the hypotheses derived from control theory and AIT, it is important to acknowledge key limitations in the data, and areas that require further research. Critically, much of the evidence marshalled was not derived from studies designed to test the current hypothesis, but from studies with other intentions. Thus, there is a need for more direct tests of the hypothesis. Moreover, there are many gaps in our relevant knowledge re level of goal/action identification and psychopathology. First, whilst there is a reasonable amount of evidence for mood disorders and anxiety disorders, other disorders including alcohol/substance abuse, eating disorders, impulse-control disorders, and psychosis are less well studied. Second, there is a key need to examine the level of goal/action identification in response to both positive and negative outcomes, and across a range of targets (e.g., positive and negative events; appetitive and aversive stimuli, familiar and unfamiliar behaviors). For example, much of the data reported for mood and anxiety disorders suggests a tendency towards a more abstract level of goal/action identification for negative events and outcomes, but it is important to determine what level of goal/action identification is utilised in response to positive events and outcomes to establish if there is a selective bias towards abstract level of identification of negative outcomes or a more general tendency to be abstract. Third, the theoretical models and emerging evidence all predict a direct effect of level of goal/action identification on delay of gratification, impulsivity, and resistance to temptation — however, to date, this is under-investigated in relevant psychological disorders, such as eating disorders, addictions, impulse-control disorders and bipolar disorder. The majority of clinical data available is relevant to examining the maladaptive effects of an abstract level of goal/action identification — for a full test of the current hypothesis, it is also necessary to test the hypothesized pathological effects of a concrete level of goal/action identification when used inappropriately.

Fourth, and critically, despite evidence that level of goal/action identification is abnormal in some disorders, the key hypothesis that the regulation of level of goal/action identification is impaired has only been directly tested in major depression. The evidence of a bias towards abstract identifications of negative concerns in GAD, PTSD, and social anxiety suggests that these disorders may also involve impaired regulation of level of goal/action identification, such that the level of goal/action identification does not become more concrete as circumstances become more difficult or mood becomes more negative. To test this possibility, a similar methodology to that used by Watkins et al. (submitted for publication) needs to be used within these anxiety disorders. Fifth, although there is some evidence in mood and anxiety disorders that level of goal/action identification predicts or influences symptoms, the direction of the causal relationship between level of goal/action identification and symptoms needs further examination across all disorders. Sixth, as noted earlier, the current analysis has focused on a particular operationalization of abstraction: whether a goal, action, or event is represented in terms of the means for how it is implemented (concrete goal/action identification and lower in control hierarchy) or in terms of its ends and consequences of why it is implemented (abstract goal/action identification and higher in control hierarchy). This operationalization is the one most consistent with the control theory approach, enables clear predictions derived from this theoretical account, and accurately reflects the majority of the evidence presented. However, this does not preclude the possibility that other conceptualizations or features of abstraction (most notably the multiple dimensions of abstraction within construal level theory, e.g., primary versus secondary characteristics, pro versus cons), may have different implications for adaptive behavior and psychological disorder than levels of goal/action identification (means versus ends), such as abstract processing facilitating the solution of certain difficult problems involving lateral thinking. However, to my knowledge, at this juncture, other than the control theory approach, there are not coherent theoretical models that predict a consistent pattern of relationships between level of abstraction and psychopathology, nor can account for the extant evidence reviewed here. Nonetheless, further examination of different operationalizations of abstraction may be warranted.

If correct, the hypothesis that dysregulation of level of goal/action identification is a transdiagnostic process contributing to different psychological disorders would have a number of important theoretical implications. First, the theoretical accounts underpinning the hypothesis of dysregulated level of goal/action identification provide a useful integrative framework that draws together a number of observations and processes in psychopathology, including repetitive thought, psychological distance, experiential avoidance, and visual perspective. For example, the conceptualization of anxiety disorders as involving a tendency towards an abstract level of goal/action identification for negative events can simultaneously account for increased pathological worry, experiential avoidance, an observer perspective, and poor problem solving, since all of these outcomes are influenced by level of goal/action identification. Second, this approach indicates the interconnectedness of these variables and predicts that manipulating one will lead to changes in another, e.g., since changes in psychological distance influence level of goal/action identification, this framework would predict differential effects on these outcomes when considering events at different temporal or spatial distance. Third, the current approach has the potential to encompass both internalizing disorders characterized by passivity (e.g., depression) and externalizing disorders characterized by impulsivity (e.g., addiction). Fourth, the focus on *regulation* of level of goal/action identification highlights the value of considering sensitivity to context and ability to shift processing in response to context as a key variable within psychopathology. This theoretical approach makes clear that a particular level of goal/action identification is not intrinsically adaptive or maladaptive but that its functionality will depend on context and flexibility. Thus, the current hypothesis is a specific operationalization of the proposal that psychological flexibility is a key dimension in psychological health, and that rigidity or “stuckness” in cognitive responses is a vulnerability factor for disorder.

The hypothesis that dysregulation of level of goal/action identification is a transdiagnostic process also has several clinical implications. First, like other transdiagnostic processes, it may have benefits for understanding the high rates of co-morbidity across psychological disorders. If as the data suggests, inflexibility in shifting level of goal/action identification with a tendency to get stuck at an abstract level of goal/action identification for negative concerns is common to mood disorders and anxiety disorders, this could, in part, account for their elevated co-morbidity, and interventions to change level of goal/action identification may reduce both anxiety and depression. Second, understanding this process may facilitate greater transfer of theoretical and treatment advances between disorders.

Third, as noted above, the current approach emphasizes a focus on function, response to context, and flexibility, rather than reifying one response as “good” and another response as “bad”. Such an approach is consistent with recent treatment interventions that emphasise adopting a functional–contextual and process perspective (e.g., acceptance and commitment therapy, ACT, Hayes, Strosahl & Wilson, 1999; behavioral activation, BA, Martell, Addis & Jacobson, 2001; rumination-focused CBT, RFCBT, Watkins et al., 2007). Approaches that utilise a functional–contextual approach may be one means to help people realize their patterns of response, and to promote the flexible use of level of goal/action identification in response to different circumstances: The development of psychological flexibility is an explicit goal of ACT; BA involves practice of adoption of alternative actions in response to difficulties, and RFCBT explicitly coaches ruminators to shift away from their predominant abstract level of goal/action identification to adopt a more concrete level of goal/action identification during self-focus. Moreover, this analysis encourages clinicians to assess how well a patient can shift their level of goal/action identification to particular circumstances and then to coach patients to correct any imbalance in their functional change in level of goal/action identification, so they can develop more flexibility in adopting the level of goal/action identification most functional for the current circumstances. The current review suggests that in some circumstances, it may be beneficial to encourage a concrete level of goal/action identification to negative outcomes (e.g., focus on how an event happened and how a goal could be pursued for depressed patients when ruminating) but in others, it may be beneficial to encourage an abstract level of goal/action identification (e.g., focus on why you may or may not implement a behavior and on its consequences and ends in response to appetitive stimuli for patients with addictions). CBT-based treatments involve techniques that may be consistent with promoting either a shift towards a concrete level of goal/action identification (e.g., detailed Socratic questioning, situational analysis, detailed activity scheduling, and breaking down tasks into steps) or towards an abstract level of goal/action identification (e.g., downward arrow technique, discussion of schemas and beliefs). Similarly, the hypothesized development of attentional control skills in mindfulness-based cognitive therapy may facilitate an individual's ability to flexibly shift level of processing in response to circumstances. Moreover, a recent therapeutic approach — The Method of Levels — is theoretically derived from control theory, and emphasizes the importance of engendering the conditions in which patients can sustain their awareness on their higher-level goals and begin to approach them differently, so as to reorganize the relationships between goals (for a fuller discussion of control theory and therapeutic change, see Higginson et al., in press).

Fourth, the literature indicates that the predominant level of goal/action identification adopted is influenced by individual differences (Vallacher & Wegner, 1989) but also by circumstances, mood, and language use. The effect of language use on level of goal/action identification is clinically important because it indicates a point for assessment and intervention. Stapel and Semin (2007) have shown that the language used by one person influences the level of goal/action identification utilised by another person. Furthermore, the language used by an individual can indicate his level of goal/action identification (Semin & Fiedler, 1988). Thus, the careful observation of language can be a means to assess level of goal/action identification, whereas the systematic use of language within therapy can be a means to influence level of goal/action identification in patients. For example, this analysis suggests the importance of using specific and concrete language with patients with depression to facilitate their shift away from abstract levels of goal/action identification (Watkins, 2009b). Likewise, multiple studies have shown that questions asking “Why?” prime the abstract level of goal/action identification, whereas questions asking “How?” prime a concrete level of goal/action identification, with accompanying functional effects (e.g., Watkins & Baracaia, 2002; Watkins et al., 2008). Building on this idea, an intervention designed to train depressed individuals to be more concrete when faced with difficulties (i.e., explicitly training a more flexible use of level of goal/action identification in response to difficulties and correcting the blocked tendency to adopt an abstract level of goal/action identification bias) involved repeated practice at asking “How?” and focusing on specific details, and reduced depression, rumination, and anxiety relative to a no-training control (Watkins & Moberly, 2009; Watkins et al., 2009). Similarly, psychological distance and visual perspective could be manipulated via imagery exercises within treatment to train participants to regulate their level of goal/action identification (e.g., Williams & Moulds, 2008).

A key issue for the current hypothesis is explaining what causes individuals with psychological disorders to adopt abnormal and maladaptive levels of goal/action identification. There are several, potentially complementary, accounts. First, just as individuals can implicitly learn over time a relationship between psychological distance and level of goal/action identification, they could also learn a relationship between context, mood state and level of goal/action identification, particularly when there is positive reinforcement for adopting a level of goal/action identification if it helps to adaptively respond to circumstances. Moreover, given the findings about the effect of language on construal, it is plausible that this learning is also shaped in childhood through socialization processes, with primary caregivers' language use encouraging adaptive shifts in construal. However, for people at risk of developing psychological disorders, these learning processes may be disrupted: an abusive, critical, or neglectful early environment would lack beneficial socialization towards appropriate use of level of goal/action identification, and in such an environment, coping responses are less likely to produce positive outcomes, making it harder to learn the functional connection between construal and context (e.g., Sarin & Nolen-Hoeksema, 2010; Spasojevic & Alloy, 2002 for evidence of early environments associated with rumination). Furthermore, abnormal regulation of level of goal/action identification may be learnt because shifts in level of goal/action identification could be negatively reinforced by the removal of distress rather than positively reinforced through goal progress. The escape theory proposes that concrete identification of sensory experience distracts from negative self-evaluations, whilst a number of theories propose that an abstract level of goal/action identification with respect to concrete reliving or recall of upsetting memories may avoid intense affect (e.g., McIsaac & Eich, 2004 re observer perspective; Martell et al., 2001; Nolen-Hoeksema, Wisco & Lyubomirsky, 2008; Watkins et al., 2007 re rumination; Williams et al., 2007 re overgeneral memories). Consistent with this, the observer perspective and rumination are associated with avoidance (Cribb et al., 2006; Kenny & Bryant, 2007; Kuyken & Moulds, 2009; Moulds, Kandris, Starr & Wong, 2007; Williams & Moulds, 2007).

Second, it may be that dysregulation of level of goal/action identification results from motivational factors. For example, the belief that it is important to understand feelings and problems would engender a tendency to adopt more abstract levels of goal/action identification (Watkins & Baracaia, 2001; Watkins & Moulds, 2005b). Third, it may be that effective regulation of level of goal/action identification depends upon the use of central executive resources (Watkins, 2008), such that deficits at effectively regulating level of goal/action identification in response to situational demands result from deficits in executive control. For example, reduced central executive resources are implicated in increased impulsive appetitive behaviors in addiction (e.g., Wiers et al., in press).

In conclusion, this paper outlines evidence for how level of goal/action identification is a potentially important dimension that influences a range of clinically-relevant outcomes including emotion, cognition, and behavior, which seems to be adaptively regulated in healthy controls. Furthermore, there is evidence that this regulation of level of goal/action identification is abnormal in a number of psychological disorders, indicating that this process is a possible transdiagnostic process.

## Figures and Tables

**Table 1:** Conditions under which different levels of prepotent goal/action identification are predicted to be adaptive versus maladaptive.

	Level of goal/action identification	
Circumstances	Abstract	Concrete
Progress toward goal at rate equal or better than standard (Neutral or Positive Affect)	Adaptive (consistency and stability of behavior)	Neutral to maladaptive (distractible by environment)
Progress toward relatively abstract goal at rate lower than standard (Negative Affect), with limited or irrelevant specification from abstract to concrete levels in goal hierarchy (i.e., Difficult, Complex, Unfamiliar), acting in service of goals	Maladaptive (procrastination, rumination, increased emotional impact)	Adaptive (problem solving, less emotional impact, initiates action)
Progress toward relatively abstract goal at rate lower than standard (negative affect), acting to avoid awareness of discrepancy	Maladaptive (as above)	Maladaptive (immersion, distraction in immediate sensation)
Progress toward relatively concrete goal at rate lower than standard, concrete goal currently unattainable	Adaptive (flexibility, substitution with alternative goal)	Maladaptive (if unable to pursue concrete goal)
Conflict between levels in hierarchy, especially when lower-level appetitive	Adaptive (self-control, delayed gratification)	Maladaptive (impulsivity, craving and immediate gratification)
Conflict resulting from incompatible sub-goals to same higher-order goal	Adaptive (flexibility, reprioritization)	Maladaptive (repetition of concrete routine)

## Footnotes

1 It is important to note that within a control theory account, goals, actions, and outcomes are effectively synonymous since the reference values (and associated perceptual input compared against the reference values) involve representations of specific behaviors (“pick up the phone”), principles of behavior (“be friendly”) and general concepts (sense of self and sense of relationship), whether performed by the individual or by others (i.e., events) and their effects on the perceived environment (i.e., outcomes). The use of the label “goal/action identification” is used here to a) acknowledge the impact of Action Identification theory and its associated evidence to the current analysis; b) make clear that levels of identification apply to representations of internally generated goals as well as actions (events) in the environment. Further, as described later, it is important to note that “level of goal/action identification” in the current article is used as shorthand to refer to the prepotent operational level at which goals, actions, events, and outcomes are currently and momentarily being represented.

2 Carver and Scheier (1990) argue that the same meta-monitoring mechanism is engaged during attempts to analyze or imagine the likelihood of different possible outcomes — such that playing through a scenario that is negative (such as when told that a task will be difficult, cf Vallacher et al., 1987) will indicate a further reduction in progress and the meta loop will yield greater doubt and negative affect.

3 The findings for the current operationalization of abstract versus concrete processing in terms of level of goal/action identification are consistent with the other features used in the broader definition in construal level theory — all of these conceptualizations of abstraction are consistently shown to increase with psychological distance. Because the current analysis focuses on predictions emerging from control theory, with its emphasis on goal hierarchy and means versus ends, a focus on other definitions of abstraction is less relevant and beyond the scope of the current article. The key point is that psychological distance influences level of goal/action identification.

## References

[bb0005] Abbott M.J., Rapee R.M. (2004). Post-event rumination and negative self-appraisal in social phobia before and after treatment. Journal of Abnormal Psychology.

[bb0010] Bandura A., Schunk D.H. (1981). Cultivating competence, self-efficacy and intrinsic interest through proximal self-motivation. Journal of Personality and Social Psychology.

[bb0015] Baumeister R.F. (1990). Suicide as escape from self. Psychological Review.

[bb0020] Baumeister R.F., Heatherton T.F. (1996). Self-regulation failure: an overview. Psychological Inquiry.

[bb0025] Bergouignan L., Lemogne C., Foucher A., Longin E., Vistoli D., Allilaire J.F. (2008). Field perspective deficit for positive memories characterizes autobiographical memory in euthymic depressed patients. Behaviour Research and Therapy.

[bb0030] Beukeboom C.J., Semin G.R. (2005). Mood and representations of behaviour: the how and why. Cognition & Emotion.

[bb0035] Beukeboom C.J., Semin G.R. (2006). How mood turns on language. Journal of Experimental Social Psychology.

[bb0040] Bickel W.K., Marsch L.A. (2001). Towards a behavioural economic understanding of drug dependence: delay discounting processes. Addiction.

[bb0045] Bless H., Clore G.L., Schwarz N., Golisano V., Rabe C., Wölk M. (1996). Mood and the use of scripts: does a happy mood really lead to mindlessness?. Journal of Personality and Social Psychology.

[bb0050] Bless H., Fiedler K., Forgas J.P. (2006). Mood and the regulation of information processing. Affect in social cognition and behavior.

[bb0055] Borkovec T.D., Ray W.J., Stober J. (1998). Worry: a cognitive phenomenon intimately linked to affective, physiological, and interpersonal behavioral processes. Cognitive Therapy and Research.

[bb0060] Borkovec T.D., Robinson E., Pruzinsky T., Depree J.A. (1983). Preliminary exploration of worry — some characteristics and processes. Behaviour Research and Therapy.

[bb0065] Bottlender M., Soyka M. (2004). Impact of craving on alcohol relapse during, and 12 months, following outpatient treatment. Alcohol and Alcoholism.

[bb0070] Broadbent D.E. (1977). Levels, hierarchies, and locus of control. Quarterly Journal of Experimental Psychology.

[bb0075] Brown J.D., Dutton K.A. (1995). The thrill of victory, the complexity of defeat: self-esteem and people's emotional reactions to success and failure. Journal of Personality and Social Psychology.

[bb0080] Brunstein J.C., Gollwitzer P.M. (1996). Effects of failure on subsequent performance: the importance of self-defining goals. Journal of Personality and Social Psychology.

[bb0085] Carver C.S. (1998). Generalization, adverse events, and development of depressive symptoms. Journal of Personality.

[bb0090] Carver C.S., Ganellen R.J. (1983). Depression and components of self-punitiveness: high standards, self-criticism, and overgeneralization. Journal of Abnormal Psychology.

[bb0095] Carver C.S., Johnson S.L. (2009). Tendencies toward mania and tendencies toward depression have distinct motivational, affective, and cognitive correlates. Cognitive Therapy and Research.

[bb0100] Carver C.S., La Voie L., Kuhl J., Ganellen R.J. (1988). Cognitive concomitants of depression: a further examination of the roles of generalization, high standards, and self-criticism. Journal of Social and Clinical Psychology.

[bb0105] Carver C.S., Scheier M.F. (1982). Control-theory — a useful conceptual-framework for personality-social, clinical, and health psychology. Psychological Bulletin.

[bb0110] Carver C.S., Scheier M.F. (1990). Origins and functions of positive and negative affect — a control-process view. Psychological Review.

[bb0115] Carver C.S., Scheier M.F. (1998). On the Self-Regulation of Behavior.

[bb0120] Clark D.M., Wells A., Heimberg R.G., Liebowitz M.R., Hope D.A., Schneier F.R. (1995). A cognitive model of social phobia. Social phobia: diagnosis, assessment, and treatment.

[bb0125] Cribb G., Moulds M.L., Carter S. (2006). Rumination and experiential avoidance in depression. Behaviour Change.

[bb0130] de Wit H. (2009). Impulsivity as a determinant and a consequence of drug use: a review of underlying processes. Addiction Biology.

[bb0135] Ehlers A., Mayou R.A., Bryant B. (1998). Psychological predictors of chronic posttraumatic stress disorder after motor vehicle accidents. Journal of Abnormal Psychology.

[bb0140] Ehlers A., Mayou R.A., Bryant B. (2003). Cognitive predictors of posttraumatic stress disorder in children: results of a prospective longitudinal study. Behaviour Research and Therapy.

[bb0145] Ehring T., Frank S., Ehlers A. (2008). The role of rumination and reduced concreteness in the maintenance of PTSD and depression following trauma. Cognitive Therapy and Research.

[bb0150] Ehring T., Szeimies A.-K., Schaffrick C. (2009). An experimental analogue study into the role of abstract thinking in trauma-related rumination. Behaviour Research and Therapy.

[bb0155] Ehring T., Watkins E.R. (2008). Repetitive negative thinking as a transdiagnostic process. International Journal of Cognitive Therapy.

[bb0160] Eisner L.R., Johnson S.L., Carver C.S. (2008). Cognitive responses to failure and success relate uniquely to bipolar depression versus mania. Journal of Abnormal Psychology.

[bb0165] Emmons R.A. (1992). Abstract versus concrete goals - personal striving level, physical illness, and psychological well-being. Journal of Personality and Social Psychology.

[bb0170] Fiedler K., Forgas J.P. (2001). Affective influences on social information processing. The handbook of affect and social cognition.

[bb0175] Field M., Schoenmakers T., Wiers R.W. (2008). Cognitive processes in alcohol binges: a review and research agenda. Current Drug Abuse Reviews.

[bb0180] Flannery B.A., Poole S.A., Gallop R.J., Volpicelli J.R. (2003). Alcohol craving predicts drinking during treatment: an analysis of three treatment instruments. Journal of Studies on Alcohol.

[bb0185] Forgas J.P. (2007). When sad is better than happy: negative affect can improve the quality and effectiveness of persuasive memories and social influence strategies. Journal of Experimental Social Psychology.

[bb0190] Forgas J.P. (2008). Affect and cognition. Perspectives in Psychological Science.

[bb0195] Forgas J.P., East R. (2008). On being happy and gullible: mood effects on scepticism and the detection of deception. Journal of Experimental Social Psychology.

[bb0200] Fujita K., Han H.A. (2009). Moving beyond deliberative control of impulses: the effect of level of construal on evaluative associations in self-control conflicts. Psychological Science.

[bb0205] Fujita K., Trope Y., Liberman N., Levin-Sagi M. (2006). Level of construal and self-control. Journal of Personality and Social Psychology.

[bb0210] Ganellen R.J. (1988). Specificity of attributions and overgeneralization in depression and anxiety. Journal of Abnormal Psychology.

[bb0215] Gasper K., Clore G.L. (2002). Attending to the big picture: mood and global versus local processing of visual information. Psychological Science.

[bb0220] Gollwitzer P.M. (1999). Implementation intentions — strong effects of simple plans. American Psychologist.

[bb0225] Gollwitzer P.M., Sheeran P. (2006). Implementation intentions and goal achievement: a meta-analysis of effects and processes. Advances in Experimental Social Psychology.

[bb0230] Hackmann A., Clark D.M., McManus F. (2000). Recurrent images and early memories in social phobia. Behaviour Research and Therapy.

[bb0235] Hamilton J.C., Greenberg J., Pyszczynski T., Cather C. (1993). A self-regulatory perspective on psychopathology and psychotherapy. Journal of Psychotherapy Integration.

[bb0240] Harvey A.G., Watkins E., Mansell W., Shafran R. (2004). Cognitive behavioural processes across psychological disorders.

[bb0245] Hayes S.C., Strosahl K., Wilson K.G. (1999). Acceptance and Commitment Therapy: An Experiential Approach to Behavior Change.

[bb0250] Hayes S.C., Wilson K.G., Gifford E.V., Follette V.M., Strosahl K. (1996). Experiential avoidance and behavioral disorders: a functional dimensional approach to diagnosis and treatment. Journal of Consulting and Clinical Psychology.

[bb0255] Heatherton T.F., Baumeister R.F. (1991). Binge eating as escape from self-awareness. Psychological Bulletin.

[bb0260] Higginson, S., Mansell, W., & Wood, A.M. (in press). An integrative mechanistic account of psychological distress, therapeutic change and recovery: the perceptual control theory approach. *Clinical Psychology Review*. doi:10.1016/j.cpr.2010.01.005. 10.1016/j.cpr.2010.01.005 20171771

[bb0265] Houser-Marko L., Sheldon K.M. (2008). Eyes on the prize or nose to the grindstone? The effects of level of goal evaluation on mood and motivation. Personality and Social Psychology Bulletin.

[bb0270] Johnson S.L., McKenzie G., McMurrich S. (2008). Ruminative responses to negative and positive affect among students diagnosed with bipolar disorder and major depressive disorder. Cognitive Therapy and Research.

[bb0275] Karniol R., Miller D.T. (1983). Why not wait? A cognitive model of self-imposed delay termination. Journal of Personality and Social Psychology.

[bb0280] Kashdan T.B., Roberts J.E. (2007). Social anxiety, depressive symptoms, and post-event rumination: affective consequences and social contextual influences. Journal of Anxiety Disorders.

[bb0285] Kavanagh D.J., Andrade J., May J. (2005). Imaginary relish and exquisite torture: the elaborated intrusion theory of desire. Psychological Review.

[bb0290] Kavanagh D.J., May J., Andrade J. (2009). Tests of the elaborated intrusion theory of craving and desire: features of alcohol craving during treatment for an alcohol disorder. British Journal of Clinical Psychology.

[bb0295] Kenny L.M., Bryant R.A. (2007). Keeping memories at an arm's length: vantage point of trauma memories. Behaviour Research and Therapy.

[bb0300] Kenny L.M., Bryant R.A., Silove D., Creamer M., O'Donnell M., McFarlane A.C. (2009). Distant memories: a prospective study of vantage point of trauma memories. Psychological Science.

[bb0305] Keough K.A., Zimbardo P.G., Boyd J.N. (1999). Who's smoking, drinking, and using drugs? Time perspective as a predictor of substance use. Basic and Applied Social Psychology.

[bb0310] Kernis M.H., Brockner J., Frankel B.S. (1989). Self-esteem and reactions to failure: the mediating role of overgeneralization. Journal of Personality and Social Psychology.

[bb0315] Kirby K.N., Petry N.M. (2004). Heroin and cocaine abusers have higher discount rates for delayed rewards than alcoholics or non-drug-using controls. Addiction.

[bb0320] Kocovski N.L., Endler N.S., Rector N.A., Flett G.L. (2005). Ruminative coping and post-event processing in social anxiety. Behaviour Research and Therapy.

[bb0325] Kuyken W., Byford S., Taylor R.S., Watkins E.R., Holden E., White K., Barrett B., Byng R., Evans A., Mullan E., Teasdale J.D. (2008). Mindfulness-based cognitive therapy to prevent relapse in recurrent depression. Journal of Consulting and Clinical Psychology.

[bb0330] Kuyken W., Moulds M.L. (2009). Remembering as an observer: how is autobiographical memory retrieval vantage perspective linked to depression?. Memory.

[bb0335] Lau G., Moulds M.L., Richardson R. (2009). Ostracism: how much it hurt depends on how you remember it. Emotion.

[bb0340] Leary M.R., Adams C.E., Tate E.B. (2006). Hypo-egoic self-regulation: exercising self-control by diminishing the influence of the self. Journal of Personality.

[bb0345] Lemogne C., Piolino P., Frizer S., Astrid C., Nathalie G.B., Roland J. (2006). Episodic autobiographical memory in depression: specificity, autonoetic consciousness, and self-perspective. Consciousness and Cognition.

[bb0350] Libby L.K., Eibach R.P., Gilovich T. (2005). Here's looking at me: the effect of memory perspective on assessments of personal change. Journal of Personality and Social Psychology.

[bb0355] Libby L.K., Shaeffer E.M., Eibach R.P. (2009). Seeing meaning in action: a bidirectional link between visual perspective and action identification level. Journal of Experimental Psychology – General.

[bb0360] Liberman N., Trope Y. (1998). The role of feasibility and desirability considerations in near and distant future decisions: a test of temporal construal theory. Journal of Personality and Social Psychology.

[bb0365] Liberman N., Trope Y., Macrae S., Sherman S. (2007). The effect of level of construal on the temporal distance of activity enactment. Journal of Experimental Social Psychology.

[bb0370] Marigold D.C., Holmes J.G., Ross M. (2007). More than words: reframing compliments from romantic partners fosters security in low self-esteem individuals. Journal of Personality and Social Psychology.

[bb0375] Markman K.D., Miller A.K. (2006). Depression, control, and counterfactual thinking: functional for whom?. Journal of Social and Clinical Psychology.

[bb0380] Martell C.R., Addis M.E., Jacobson N.S. (2001). Depression in Context: Strategies for Guided Action.

[bb0385] Martin L.L., Tesser A., Uleman J.S., Bargh J.A. (1989). Toward a motivational and structural theory of ruminative thought. Unintended thought.

[bb0390] Martin L.L., Tesser A., Wyer R.S. (1996). Some ruminative thoughts. Ruminative thoughts.

[bb0395] McCrea S.M., Liberman N., Trope Y., Sherman S.J. (2008). Level of construal and procrastination. Psychological Science.

[bb0400] McIsaac H.K., Eich E. (2004). Vantage point in traumatic memory. Psychological Science.

[bb0405] Mellings T.M.B., Alden L.E. (2000). Cognitive processes in social anxiety: the effects of self-focus, rumination and anticipatory processing. Behaviour Research and Therapy.

[bb0410] Miller W.R. (1998). Why do people change addictive behavior?. Addiction.

[bb0415] Moberly N.J., Watkins E.R. (2006). Processing mode influences the relationship between trait rumination and emotional vulnerability. Behavior Therapy.

[bb0420] Moulds M.L., Kandris E., Starr S., Wong A.C.M. (2007). The relationship between rumination, avoidance and depression in a non-clinical sample. Behaviour Research and Therapy.

[bb0425] Muraven M., Collins R.L., Neinhaus K. (2002). Self-control and alcohol restraint: an initial application of the self-control strength model. Psychology of Addictive Behavior.

[bb0430] Neumann A., Philippot P. (2007). Specifying what makes a personal memory unique enhances emotion regulation. Emotion.

[bb0435] Nolen-Hoeksema S. (2000). The role of rumination in depressive disorders and mixed anxiety/depressive symptoms. Journal of Abnormal Psychology.

[bb0440] Nolen-Hoeksema S., Harrell Z.A. (2002). Rumination, depression, and alcohol use: tests of gender differences. Journal of Cognitive Psychotherapy.

[bb0445] Nolen-Hoeksema S., Stice E., Wade E., Bohon C. (2007). Reciprocal relations between rumination and bulimic, substance abuse, and depressive symptoms in female adolescents. Journal of Abnormal Psychology.

[bb0450] Nolen-Hoeksema S., Wisco B.E., Lyubomirsky S. (2008). Rethinking rumination. Perspectives on Psychological Science.

[bb0455] Pahwal P., Hyman S.M., Sinha R. (2008). Craving predicts time to cocaine relapse: further validation of the now and brief versions of the cocaine craving questionnaire. Drug and Alcohol Dependence.

[bb0460] Petry N.M. (2001). Delay discounting of money and alcohol in actively using alcoholics, currently abstinent alcoholics, and controls. Psychopharmacology.

[bb0465] Pham L.B., Taylor S.E. (1999). From thought to action: effects of process-versus outcome-based mental simulations on performance. Personality and Social Psychology Bulletin.

[bb0470] Philippot P., Baeyens C., Douilliez C. (2006). Specifying emotional information: regulation of emotional intensity via executive processes. Emotion.

[bb0475] Philippot P., Schaefer A., Herbette G. (2003). Consequences of specific processing of emotional information: impact of general versus specific autobiographical memory priming on emotion elicitation. Emotion.

[bb0480] Powers W.T. (1973). Behavior: The Control of Perception.

[bb0485] Powers W.T. (1973). Feedback: beyond behaviorism. Science.

[bb0490] Pyszczynski T., Greenberg J. (1987). Self-regulatory perseveration and the depressive self-focusing style: a self-awareness theory of reactive depression. Psychological Bulletin.

[bb0495] Raes F., Hermans D., Williams J.M.G., Eelen P. (2006). Reduced autobiographical memory specificity and affect regulation. Cognition and Emotion.

[bb0500] Rapee R.M., Heimberg R.G. (1997). A cognitive–behavioral model of anxiety in social phobia. Behaviour Research and Therapy.

[bb0505] Rimes K.A., Watkins E. (2005). The effects of self-focused rumination on global negative self-judgements in depression. Behaviour Research and Therapy.

[bb0510] Rivkin I.D., Taylor S.E. (1999). The effects of mental simulation on coping with controllable stressful events. Personality and Social Psychology Bulletin.

[bb0515] Rossow I. (2008). Alcohol consumption and discounting. Addiction Research and Theory.

[bb0520] Sarin S., Nolen-Hoeksema S. (2010). The dangers of dwelling: an examination of the relationship between rumination and consumptive coping in survivors of childhood sexual abuse. Cognition and Emotion.

[bb0525] Schmeichel B.J., Vohs K. (2009). Self-affirmation and self-control: affirming core values counteracts ego depletion. Journal of Personality and Social Psychology.

[bb0530] Semin G.R., Fiedler K. (1988). The cognitive functions of linguistic categories in describing persons: social cognition and language. Journal of Personality and Social Psychology.

[bb0535] Spasojevic J., Alloy L.B. (2002). Who becomes a depressive ruminator? Developmental Antecedents of ruminative response style. Journal of Cognitive Psychotherapy: An International Quarterly.

[bb0540] Spasojević J., Alloy L.B. (2001). Rumination as a common mechanism relating depressive risk factors to depression. Emotion.

[bb0545] Spurr J.M., Stopa L. (2003). The observer perspective: effects on social anxiety and performance. Behaviour Research and Therapy.

[bb0550] Stapel D.A., Semin G.R. (2007). The magic spell of language: linguistic categories and their perceptual consequences. Journal of Personality and Social Psychology.

[bb0555] Steele C.M., Josephs R.A. (1990). Alcohol myopia: its prized and dangerous effects. American Psychologist.

[bb0560] Stöber J. (1998). Worry, problem elaboration and suppression of imagery: the role of concreteness. Behaviour Research and Therapy.

[bb0565] Stöber J., Borkovec T.D. (2002). Reduced concreteness of worry in generalized anxiety disorder: findings from a therapy study. Cognitive Therapy and Research.

[bb0570] Stöber J., Tepperwien S., Staak M. (2000). Worrying leads to reduced concreteness of problem elaborations: evidence for the avoidance theory of worry. Anxiety Stress and Coping.

[bb0575] Stock J., Cervone D. (1990). Proximal goal-setting and self-regulatory processes. Cognitive Therapy and Research.

[bb0580] Storbeck J., Clore G.L. (2005). With sadness comes accuracy; with happiness, false memory: mood and the false memory effect. Psychological Science.

[bb0585] Takano K., Tanno Y. (2010). Concreteness of thinking and self-focus. Consciousness and Cognition.

[bb0590] Taylor S.E., Pham L.B., Rivkin I.D., Armor D.A. (1998). Harnessing the imagination — mental simulation, self-regulation, and coping. American Psychologist.

[bb0595] Teasdale J.D., Segal Z.V., Williams J.M.G., Ridgeway V.A., Soulsby J.M., Lau M.A. (2000). Prevention of relapse/recurrence in major depression by mindfulness-based cognitive therapy. Journal of Consulting and Clinical Psychology.

[bb0600] Trope Y., Liberman N. (2003). Temporal construal. Psychological Review.

[bb0605] Trope Y., Liberman N., Wakslak C. (2007). Level of goal and action identifications and psychological distance: effects on representation, prediction, evaluation, and behavior. Journal of Consumer Psychology.

[bb0610] Vallacher R.R., Wegner D.M. (1987). What do people think they're doing? Action identification and human behavior. Psychological Review.

[bb0615] Vallacher R.R., Wegner D.M. (1989). Levels of personal agency: individual variation in action identification. Journal of Personality and Social Psychology.

[bb0620] Vallacher R.R., Wegner D.M., Frederick J. (1987). The presentation of self through action identification. Social Cognition.

[bb0625] Vallacher R.R., Wegner D.M., McMahan S.C., Cotter J., Larsen K.A. (1992). On winning friends and influencing people: action identification and self-presentation success. Social Cognition.

[bb0630] Vallacher R.R., Wegner D.M., Somoza M.P. (1989). That's easy for you to say: action identification and speech fluency. Journal of Personality and Social Psychology.

[bb0635] Vassilopoulos S., Watkins E.R. (2009). Adaptive and maladaptive self-focus: a pilot extension study with individuals high and low in fear of negative evaluation. Behavior Therapy.

[bb0640] Vohs K.D., Heatherton T.F. (2000). Self-regulatory failure: a resource-depletion approach. Psychological Science.

[bb0645] Vohs K.D., Schmeichel B.J. (2003). Self-regulation and the extended now: controlling the self alters the subjective experience of time. Journal of Personality and Social Psychology.

[bb0650] Vuchinich R.E., Simpson C.A. (1998). Hyperbolic temporal discounting in social drinkers and problem drinkers. Experimental and Clinical Psychopharmacology.

[bb0655] Wakslak C., Trope Y. (2009). The effect of construal level on subjective probability estimates. Psychological Science.

[bb0660] Wakslak C.J., Trope Y., Liberman N., Alony R. (2006). Seeing the forest when entry is unlikely: probability and the mental representation of events. Journal of Experimental Psychology: General.

[bb0665] Watkins E. (2004). Adaptive and maladaptive ruminative self-focus during emotional processing. Behaviour Research and Therapy.

[bb0670] Watkins E.R. (2008). Constructive and unconstructive repetitive thought. Psychological Bulletin.

[bb0675] Watkins E.R. (2009). Depressive rumination and co-morbidity: evidence for brooding as a transdiagnostic process. Journal of Rational-Emotive and Cognitive-Behavior Therapy.

[bb0680] Watkins, E. R. (2009b). Depressive rumination: investigating mechanisms to improve cognitive–behavioral treatments. *Cognitive Behaviour Therapy, 38*(1), 8–14. 10.1080/16506070902980695 PMC3357968 19697180

[bb0685] Watkins E.R., Baeyens C.B., Read R. (2009). Concreteness training reduces dysphoria: proof-of-principle for repeated cognitive bias modification in depression. Journal of Abnormal Psychology.

[bb0690] Watkins E., Baracaia S. (2001). Why do people ruminate in dysphoric moods?. Personality and Individual Differences.

[bb0695] Watkins E., Baracaia S. (2002). Rumination and social problem-solving in depression. Behaviour Research and Therapy.

[bb0700] Watkins E.R., Moberly N.J. (2009). Concreteness training reduces dysphoria: a pilot proof-of-principle study. Behaviour Research and Therapy.

[bb0705] Watkins E.R., Moberly N.J., Moulds M.L. (2008). Processing mode causally influences emotional reactivity: distinct effects of abstract versus concrete construal on emotional response. Emotion.

[bb0710] Watkins E., Moulds M. (2005). Distinct modes of ruminative self-focus: impact of abstract versus concrete rumination on problem solving in depression. Emotion.

[bb0715] Watkins E., Moulds M. (2005). Positive beliefs about rumination in depression: a replication and extension. Personality and Individual Differences.

[bb0720] Watkins E., Moulds M.L. (2007). Reduced concreteness of rumination in depression: a pilot study. Personality and Individual Differences.

[bb0725] Watkins E., Scott J., Wingrove J., Rimes K., Bathurst N., Steiner H. (2007). Rumination-focused cognitive behaviour therapy for residual depression: a case series. Behaviour Research and Therapy.

[bb0730] Watkins E., Teasdale J.D. (2001). Rumination and overgeneral memory in depression: effects of self-focus and analytic thinking. Journal of Abnormal Psychology.

[bb0735] Watkins E., Teasdale J.D. (2004). Adaptive and maladaptive self-focus in depression. Journal of Affective Disorders.

[bb0740] Watkins, E.R., Moberly, N.J., and Moulds, M.L. (submitted for publication). When the ends outweigh the means: mood and level-of-construal in depression. 10.1080/02699931.2010.532389 PMC3471317 22017614

[bb0745] Webb T.L., Sheeran P. (2003). Can implementation intentions help to overcome ego-depletion?. Journal of Experimental Social Psychology.

[bb0750] Wegner D.M., Vallacher R.R. (1987). The trouble with action. Social Cognition.

[bb0755] Wegner D.M., Vallacher R.R., Dizadji D. (1989). Do alcoholics know what they're doing: identifications in the act of drinking. Basic and Applied Social Psychology.

[bb0760] Wegner D.M., Vallacher R.R., Kiersted G.W., Dizadji D. (1986). Action identification in the emergence of social behavior. Social Cognition.

[bb0765] Wegner D.M., Vallacher R.R., Macomber G., Wood R., Arps K. (1984). The emergence of action. Journal of Personality and Social Psychology.

[bb0770] Weller R.E., Cook E.W., Avsar K.B., Cox J.E. (2008). Obese women show greater delay discounting than healthy weight women. Appetite.

[bb0775] Wells A., Clark D.M., Ahmad S. (1998). How do I look with my minds eye: perspective taking in social phobic imagery. Behaviour Research and Therapy.

[bb0780] Wells A., Papageorgiou C. (1999). The observer perspective: biased imagery in social phobia, agoraphobia, and blood injury phobia. Behaviour Research and Therapy.

[bb0785] Wenzlaff R.M., Grozier S.A. (1988). Depression and the magnification of failure. Journal of Abnormal Psychology.

[bb0790] Wiers, R.W., Field, M., and Stacy, A.W. (in press). Passion's slave? Cognitive processes in alcohol and drug abuse. In K. Sher (Ed.) *Oxford Handbook of Substance Use Disorders*. Oxford: Oxford University Press.

[bb0795] Williams J.M.G., Barnhofer T., Crane C., Hermans D., Raes F., Watkins E., Dalgleish T. (2007). Autobiographical memory specificity and emotional disorder. Psychological Bulletin.

[bb0800] Williams A.D., Moulds M.L. (2007). Cognitive avoidance of intrusive memories: recall vantage perspective and associations with depression. Behaviour Research and Therapy.

[bb0805] Williams A.D., Moulds M.L. (2008). Manipulating recall vantage perspective of intrusive memories in dysphoria. Memory.

[bb0810] Wong Q.J.J., Moulds M.L. (2009). Impact of rumination versus distraction on anxiety and maladaptive self-beliefs in socially anxious individuals. Behaviour Research and Therapy.

[bb0815] Zimbardo P.G., Boyd J.N. (1999). Putting time in perspective: a valid, reliable individual difference metric. Journal of Personality and Social Psychology.

[bb0820] Zimbardo P.G., Keough K.A., Boyd J.N. (1997). Present time perspective as a predictor of risky driving. Personality and Individual Differences.

